# An Evolutionary Study of *Carex* Subg. *Psyllophorae* (Cyperaceae) Sheds Light on a Strikingly Disjunct Distribution in the Southern Hemisphere, With Emphasis on Its Patagonian Diversification

**DOI:** 10.3389/fpls.2021.735302

**Published:** 2021-11-08

**Authors:** Carmen Benítez-Benítez, Ana Otero, Kerry A. Ford, Pablo García-Moro, Sabina Donadío, Modesto Luceño, Santiago Martín-Bravo, Pedro Jiménez-Mejías

**Affiliations:** ^1^Botany Area, Department of Molecular Biology and Biochemical Engineering, Universidad Pablo de Olavide, Seville, Spain; ^2^Grainger Bioinformatics Center, Department of Science and Education, The Field Museum, Chicago, IL, United States; ^3^Allan Herbarium, Manaaki-Whenua Landcare Research, Lincoln, New Zealand; ^4^Department of Biology (Botany), Universidad Autónoma de Madrid, Madrid, Spain; ^5^Centro de Investigación en Biodiversidad y Cambio Global (CIBC-UAM), Universidad Autónoma de Madrid, Madrid, Spain; ^6^Instituto de Botánica Darwinion (ANCEFN-CONICET), San Isidro, Argentina

**Keywords:** Andes, biogeography, disjunction, Gondwana, long-distance dispersal, niche conservatism

## Abstract

*Carex* subgenus *Psyllophorae* is an engaging study group due to its early diversification compared to most *Carex* lineages, and its remarkable disjunct distribution in four continents corresponding to three independent sections: sect. *Psyllophorae* in Western Palearctic, sect. *Schoenoxiphium* in Afrotropical region, and sect. *Junciformes* in South America (SA) and SW Pacific. The latter section is mainly distributed in Patagonia and the Andes, where it is one of the few *Carex* groups with a significant *in situ* diversification. We assess the role of historical geo-climatic events in the evolutionary history of the group, particularly intercontinental colonization events and diversification processes, with an emphasis on SA. We performed an integrative study using phylogenetic (four DNA regions), divergence times, diversification rates, biogeographic reconstruction, and bioclimatic niche evolution analyses. The crown age of subg. *Psyllophorae* (early Miocene) supports this lineage as one of the oldest within *Carex*. The diversification rate probably decreased over time in the whole subgenus. Geography seems to have played a primary role in the diversification of subg. *Psyllophorae*. Inferred divergence times imply a diversification scenario away from primary Gondwanan vicariance hypotheses and suggest long-distance dispersal-mediated allopatric diversification. Section *Junciformes* remained in Northern Patagonia since its divergence until Plio-Pleistocene glaciations. Andean orogeny appears to have acted as a northward corridor, which contrasts with the general pattern of North-to-South migration for temperate-adapted organisms. A striking niche conservatism characterizes the evolution of this section. Colonization of the SW Pacific took place on a single long-distance dispersal event from SA. The little ecological changes involved in the trans-Pacific disjunction imply the preadaptation of the group prior to the colonization of the SW Pacific. The high species number of the section results from simple accumulation of morphological changes (disparification), rather than shifts in ecological niche related to increased diversification rates (radiation).

## Introduction

The Neotropic is one of the most biodiverse regions, containing seven of the 36 currently recognized biodiversity hotspots of Earth (Koenig, 2016), and is considered an evolutionary hub for research about the origin of biological diversity (Rull, 2008). Most available phylogeographical studies focusing on plants along South America (SA) have been primarily restricted to the Tropical Andes region (Muellner et al., 2005; Marcheli and Gallo, 2006; Acosta and Premoli, 2010; Table 1), while Patagonia remains among the least phylogeographically studied regions of SA despite having been affected more than other areas of the continent by historical environmental changes, both recently and over geological time scales (Rabassa, 2008; Sérsic et al., 2011). The interest in understanding the historical processes impacting Patagonian biota has been increasing in the last decades, with a special focus on animals (e.g., rodents, Kim et al., 1998; lizards, Morando et al., 2004, 2007; Breitman et al., 2012; fishes, Cussac et al., 2004; Ruzzante et al., 2008; birds, Calderón et al., 2014; Cadena et al., 2020). However, understanding of the evolutionary history and processes of plant diversification in this region has not bloomed until recently (Azpilicueta et al., 2009; Jakob et al., 2009; Tremetsberger et al., 2009; Cosacov et al., 2010; Sede et al., 2012; Nicola et al., 2014, 2019; Soliani et al., 2015; López and Bonasora, 2017; Table 1).

**Table 1:** Review of current South American phylogeographical knowledge of plants based on published articles with a focus on the Andes and Patagonia regions.

**Taxa**	**Distribution**	**Methodology**	**Main conclusions**	**References**
*Anarthrophyllum desideratum* (Fabaceae)	Southern Patagonia	Plastid haplotypes	Survival in refugia during Pleistocene glaciations	Cosacov et al., 2013
*Austrocedrus* (Cupressaceae)	Andean-Patagonian forests	Nuclear microsatellites and isozyme loci; SDMs projections to LGM	Survival during Pleistocene glaciations in several small refuges along the Andes in xeric environments with southwards post-glacial colonizations.	Pastorino et al., 2004; Souto et al., 2015
*Calceolaria polyrhiza* (Calceolariaceae)	Southern Andes and Patagonian steppe	Plastid haplotypes	Survival during Pleistocene glaciations in multiple refugia and multiple postglacial colonization routes. Impacts of Mio-Pliocene geoclimatic events in the genetic structure.	Cosacov et al., 2010
*Chuquiraga* (Asteraceae)	Andes and Patagonia	Morphological characters	Evolutionary radiations because of geo-climatic changes since Andean orogeny to Pleistocene-Holocene fluctuations.	Ezcurra, 2002
*Embothrium* (Proteaceae)	Temperate forests in Patagonia	Plastid haplotypes and isozyme loci	*In-situ* survival during Pleistocene glaciations (northern and southern ranges) with post-glacial colonizations from multiple refugia.	Souto and Premoli, 2007; Vidal-Russell et al., 2011
*Escallonia* (Escalloniaceae)	Andes	Plastid haplotypes and AFLPs	Strong pattern of genetic, morphological and geographical differentiation associated with ancient Andean orogeny.	Morello and Sede, 2016
*Eucryphia cordifolia* (Cunoniaceae)	Andes and southern Patagonia	Plastid haplotypes	Survival during Pleistocene glaciations in several refugia.	Segovia et al., 2012
*Fitzroya cupressoides* (Cupressaceae)	Southern Andes and Patagonia	Isozyme loci	Survival during Pleistocene glaciations in multiple refugia with post-glacial northernwards colonizations.	Premoli et al., 2000
*Heliotropium* (Heliotropiaceae)	Andes	Plastid haplotypes and ITS	Rapid diversification of the main lineages of the genus during late Miocene-early Pliocene in response to Andean uplift and xeric environments.	Luebert et al., 2011
*Herbertus* (Herbertaceae)	Southern Patagonia	Plastid haplotypes and ITS	Strong influence of the Andean uplift limiting gene flow and generating new environmental conditions which seem to allow dispersals.	He and Sun, 2017
*Hordeum* (Poaceae)	Southern Andes, Patagonia and Tierra del Fuego	Plastid haplotypes	Survival during Pleistocene glaciations within their distribution range and post-glacial colonization of southern habitats.	Jakob et al., 2009
*Hypericum* (Hypericaceae)	High Andean grasslands	ITS	High morphological diversity with low genetic differentiation because of hybridization, incomplete lineage sorting or adaptive radiation.	Nürk et al., 2013
*Hypochaeris* (Asteraceae)	Southern Andes, Patagonia and Tierra de Fuego	Plastid haplotypes and AFLPs	Survival during Pleistocene glaciations within its distribution range and rapid post-glacial expansions from close refugia.	Muellner et al., 2005; Tremetsberger et al., 2009
*Lupinus* (Leguminosae)	Andes	ITS and regulatory gene	Plio-Pleistocene colonization of new environments because of Andean uplift.	Hughes and Eastwood, 2006
*Mulinum spinosum* (Apiaceae)	Southern Andes and Patagonian steppe	Plastid haplotypes	*In-situ* survival during Pleistocene glaciations but with little impact in phylogeographic structure.	Sede et al., 2012
*Nassauvia* (Asteraceae)	Southern Andes and Patagonian steppe	Plastid haplotypes and ITS; SDMs projected onto LGM.	Survival during Pleistocene glaciations in refugia isolating northern from southern populations, with posterior range expansion to steppe. Strong genetic structure promoted by the Andean uplift.	Nicola et al., 2014, 2019
*Nothofagus* (Nothofagaceae)	Temperate forests in the Andes, Patagonia and Tierra del Fuego	Plastid haplotypes, nuclear and chloroplast DNA microsatellites, isozyme marker loci; and SDMs projected onto LGM	Survival during Pleistocene glaciations in several refugia and post-glacial colonizations from northernmost shelters. Strong genetic structure promoted by recent glacial-interglacial periods, as well as by ancient events.	Marcheli and Gallo, 2006; Azpilicueta et al., 2009; Pastorino et al., 2009; Mathiasen and Premoli, 2010; Premoli et al., 2010; Soliani et al., 2015
*Oxalis* (Oxalidaceae)	Southern Andes-Patagonia (including coast)	Plastid haplotypes, ITS, and ISSR markers; and SDMs	*In-situ* survival during Pleistocene glaciations in several refugia, with diversification mostly promoted by Andean uplift; lineages preadapted to xeric environments.	Heibl and Renner, 2012; López and Bonasora, 2017
*Podocarpus nubigena* (Podocarpaceae)	Southern Andes	Isozyme marker loci	Survival during Pleistocene glaciations and post-glacial southwards colonizations.	Quiroga and Premoli, 2010
*Prosopis chilensis* (Fabaceae)	Northern Patagonia and Central Andes	Plastid haplotypes and ITS	Survival *in-situ* of lineages vs. recent colonization of others. Morphological differences promoted by geographical isolation and local adaptations.	Aguilar et al., 2020
*Silene* sect. *Physolychnis* (Caryophyllaceae)	Andes and Patagonian steppe	Plastid haplotypes and ITS	Plio-Pleistocene migration from North America with *in-situ* diversification in South America.	Frajman et al., 2018
*Valeriana* (Valerianaceae)	Southern Andes	Plastid haplotypes and ITS	*In-situ* survival and high genetic diversity as consequence of multiple radiations colonizing new habitats.	Bell et al., 2012

Different biogeographic affinities among SA and other landmasses (intercontinental disjunctions) have previously been described. One of the most intensely studied disjunctions has been the amphitropical pattern (including the bipolar disjunction). This involves taxa distributed at medium and high latitudes of both the hemispheres. It has mainly been explained through long-distance dispersal (LDD) likely by birds and dated back to cold periods of the Plio-Pleistocene (Simpson et al., 2005, 2017; Spalik et al., 2010; Villaverde et al., 2017a). Another less explored pattern is the trans-Caribbean disjunction. It is observed in organisms distributed in regions adjacent to the northern and southern shores of the Caribbean Sea. It has been associated with bird dispersal through the American-Atlantic flyway (Jiménez-Mejías et al., 2018, 2020). Another very striking disjunction pattern is that involving SA with New Zealand (NZ), the often so-called Gondwanan disjunction. In the 19th century, Hooker (1853) and Darwin (1859) discussed vicariance vs. dispersal-based explanations for organisms displaying disjunct distributions among SA, NZ, and also Africa (McLoughlin, 2001; Givnish et al., 2004; Specht, 2006). On the one hand, such distributions were often attributed to vicariance enabled by the tectonic dynamics of Gondwanan landmasses (Biffin et al., 2010; Noben et al., 2017). On the other hand, recent molecular dating studies have confirmed that LDD explains some of the Gondwanan distribution patterns better than vicariance-like scenarios, especially in plants (McGlone et al., 2001; Sanmartín and Ronquist, 2004). Accordingly, it has been shown that some species have reached both sides of the Southern Pacific (SA and NZ) by direct LDD in relatively recent times (Knapp et al., 2005; Otero et al., 2019). Alternatively, migration between these landmasses *via* stepping-stones across Antarctica has also been argued (de la de la Estrella et al., 2019). Just a few groups of ancient (Cretaceous) origin seem to have Gondwanan distributions explained by tectonic vicariance, such as Araucariaceae (Biffin et al., 2010; Kranitz et al., 2014).

Within SA, the Andean orogenesis had a great impact on species distribution (Antonelli et al., 2009). However, it was a slow uneven process, with the northern and southern parts of the range undergoing uplift at remarkably different times (middle Eocene-early Oligocene and Miocene-Pleistocene, respectively; Antonelli et al., 2009; Mora et al., 2020). Phylogeographic studies have shown that this mountain range acted on the one hand as a dispersal route in some groups of plants (Bell and Donoghue, 2005; Nürk et al., 2013), and on the other hand, as a barrier against vicariance triggering rapid diversification (Hughes and Eastwood, 2006). At the southernmost part of the continent, Patagonia was also dramatically affected by several geoclimatic events during the Cenozoic. The widening of the Drake Passage between the Southern Cone and Antarctica (49–17 million years ago, mya) caused changes in the ocean circulation intensifying the circum-Antarctic current, freezing Antarctica (ca. 30 mya; Cantrill and Poole, 2012), and subsequently cooling the surrounding landmasses. This may have facilitated the establishment and diversification of species with more cold-temperate climate preferences. From the late Oligocene to early Miocene, the shorelines of Patagonia underwent repeated shifting, continually creating new environmental opportunities, with the flatlands being repeatedly flooded by the sea in the form of marine transgressions and promoting the fragmentation of these areas (Encinas et al., 2018). At the end of the Cenozoic, the previously mentioned Andean orogeny in Patagonia introduced a cold and dry climate toward the east promoting the replacement of Miocene subtropical savannas by arid steppes, with taxa adapted to cooler xeric environments (tussock grasses and shrubs; Barreda and Palazzesi, 2007; Sede et al., 2012). This event also contributed to the retreat of ancient forests toward the more humid Andean slopes after the Patagonian desertification (Rabassa, 2008). In more recent times, different glaciations and associated climatic events have affected the distribution and divergence of species since the late Miocene (Ramos and Ghiglione, 2008; Martínez and Kutschker, 2011; Ponce et al., 2011; Breitman et al., 2012). The oldest known Patagonian glaciation took place around the Mio-Pliocene boundary (5–7 mya; Rabassa et al., 2005). This was subsequently followed by the large late Pliocene glaciations (ca. 3.5 mya; Rabassa et al., 2005). Later, the Quaternary glacial-interglacial cycles finished shaping present species distribution patterns (Markgraf et al., 1995; Hewitt, 2000; Rabassa, 2008). The most important was the Greatest Patagonian Glaciation (1–1.2 mya), exclusive to the Southern Cone, involving a large expansion of ice sheets in the Patagonian steppe. The Last Glacial Maximum (LGM, c. 18-21 ka) also influenced Patagonia and took place there and in the Northern Hemisphere, simultaneously (Rabassa et al., 2011). The absence of a continuous ice sheet during the LGM in southern SA and the occurrence of interglacial periods are associated with the movement of species following a more stable climate and the presence of refugia mostly north and east (Markgraf, 1983; Markgraf et al., 1995; Holderegger and Thiel-Egenter, 2009; Ponce et al., 2011). However, other studies point to several species presenting a reversed pattern, with refugia in higher (southern) latitudes with postglacial recolonizations to the north (Jakob et al., 2009; Tremetsberger et al., 2009; Cosacov et al., 2010; Sérsic et al., 2011; Breitman et al., 2012; Sede et al., 2012; Vera-Escalona et al., 2012; Frajman et al., 2018), which contrast with what is documented for the Northern Hemisphere (Hewitt, 2004). Even species endemic to Southern Patagonia (S Patagonia; incl. Tierra del Fuego) seem to have had glacial refugia exclusively in that same area during the Pleistocene (Muellner et al., 2005).

To illustrate the biogeographic history of this region and the implied speciation patterns, it is important to understand how geo-climatic events have interacted with the ecological requirements of plant species and how this has affected the evolution of lineages through time. Some species unable to adapt to new environmental conditions are forced to move in search of an environment with similar conditions to their original requirements (niche conservatism; Wiens and Graham, 2005; Kozak and Wiens, 2006; Losos, 2008; Wiens et al., 2010), while others are able to adapt to new environmental conditions, persisting in their original niche as the conditions change, or colonizing newly available habitats (niche shifts; Pearman et al., 2008; Spalink et al., 2016). Despite the undoubted role of geography and climate shaping distribution patterns in SA, the change in environmental niche seems to have also played a role in the diversification of species at more local and regional scales (Aguilar et al., 2020; Luebert et al., 2020). That is to say, geographical isolation could have triggered divergence of disjunct populations and eventual speciation *via* local adaptation (Frajman et al., 2018; Aguilar et al., 2020).

**Figure 1:**
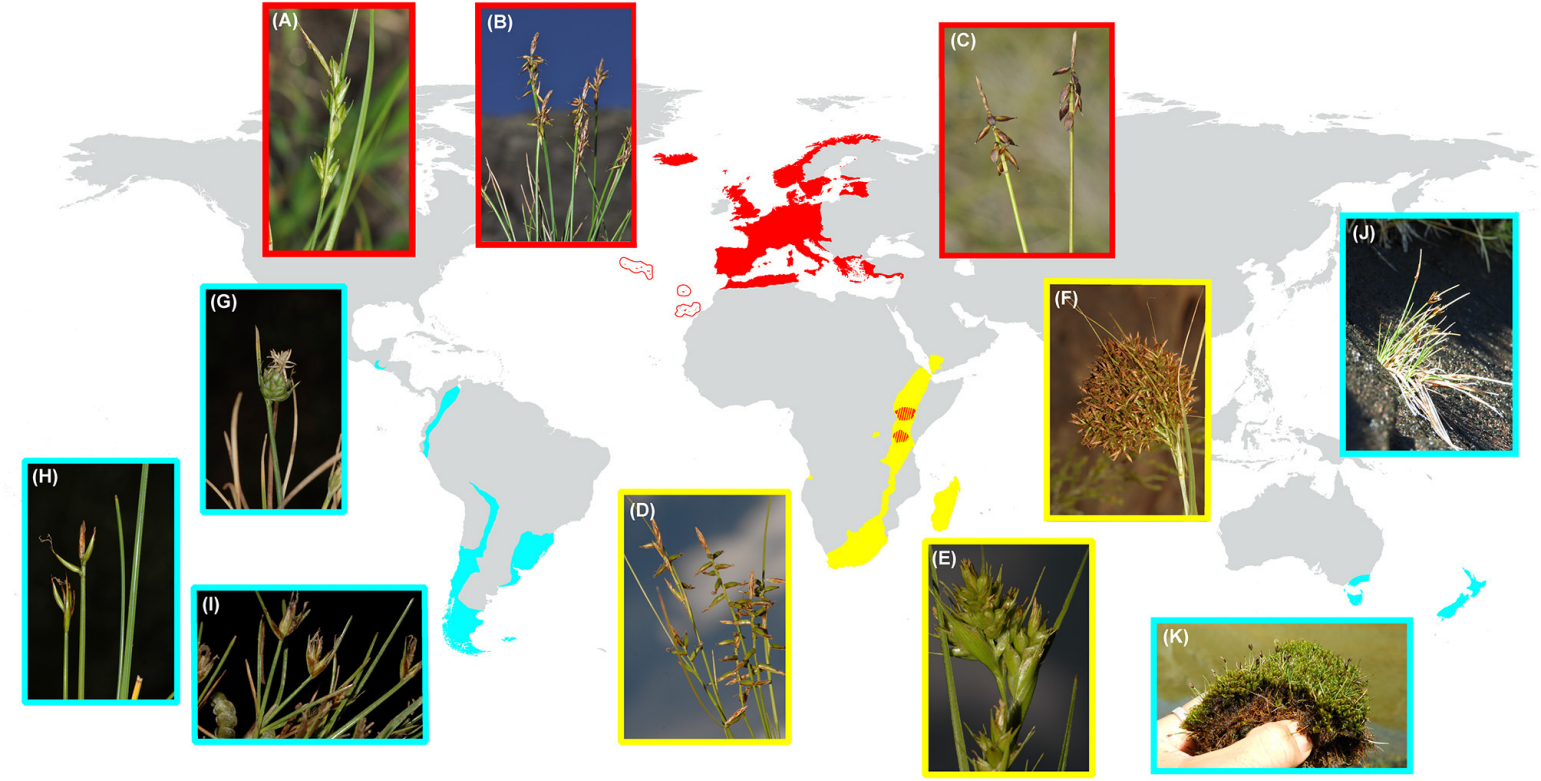
Distribution map of *Carex* subg. *Psyllophorae* including its three sections: (1) *Psyllophorae* in Western Palearctic; (2) *Schoenoxiphium* in the Afrotropical region; and (3) *Junciformes* in SA and SW Pacific. The colors of each section are according to Figure 2A (AAR analysis). Photographs display the morphological variation in each section: (**A–C** sect. *Psyllophorae*) **(A)** *C. distachya* Desf., Spain, Ávila, Arenas de S. Pedro, **(B)** *C. macrostyla* Lapeyr., Spain, Lerida, Aran Valley, **(C)** *C. pulicaris* L., W Iceland; (**D–F** sect. *Schoenoxiphium*), **(D)** *C. killickii* Nelmes, Lesotho, Roma-Semonkong, **(E)** *C. dregeana* Kunth, South Africa, Drakensberg, Cathedral Peak, **(F)** *C. multispiculata* Luceño and Martín-Bravo, South Africa, Kwazulu-Natal, Cathedral Peak; (**G–K** sect. *Junciformes*) **(G)** *C. andina* Phil, Chile, Santiago, Nevado Valley, **(H)** *C. camptoglochin* V. I. Krecz., Chile, Punta Arenas, **(I)** *C. vallis-pulchrae* var. *barrosiana* G. A. Wheeler, Argentina, Tierra del Fuego, Ushuaia, **(J)** *C. acicularis* Boott, NZ, Eyre mountains, **(K)** *C. enysii* Petrie, NZ, Harris mountains. AAR, ancestral area reconstruction, NZ, New Zealand.

*Carex* L. (Cyperaceae) is one of the most diverse and widely distributed plant groups on Earth (ca. 2000 spp; Egorova, 1999; Ball and Reznicek, 2002), with a cosmopolitan distribution and only absent from Antarctica and a few tropical areas (Roalson et al., 2021). It has its highest species richness in cold-temperate regions of the Northern Hemisphere and inhabits a great diversity of habitats (Martín-Bravo et al., 2019). Subgenus *Psyllophorae* (Degl.) Peterm. is one of the main lineages of the genus *Carex* (Villaverde et al., 2020), but with a relatively small number of species (56 species according to Roalson et al., 2021) compared to other subgenera [*Carex, Vignea* (P. Beauv. ex T. Lestib.) Peterm.; >500 species, Roalson et al., 2021]. It displays a striking disjunct distribution (Figure 1) as is present in the Western Palearctic (sect. *Psyllophorae* Degl.), Afrotropical region (sect. *Schoenoxiphium* Nees), as well as SA and SW Pacific [sect. Junciformes (Boeckeler) Kük]. A fourth group (Curvula-clade) has been included in this subgenus but its placement still needs further study because of the extremely short branch supporting the group (Roalson et al., 2021). Subgenus *Psyllophorae* has been dated back to the late Oligocene with relatively deep nodes (Martín-Bravo et al., 2019). The group as a whole seems to match two remarkable distribution patterns. On the one hand, a Rand-Flora pattern (Mairal et al., 2017) is drawn between the Western Palearctic (sect. *Psyllophora*) and Afrotropical (sect. *Schoenoxiphium*) distribution. On the other hand, a reminiscent pattern of Gondwanan distribution (Sanmartín and Ronquist, 2004) is suggested for the circum-Antarctic disjunction between SA and SW Pacific (sect. *Junciformes*), and Tropical Africa (sect. *Schoenoxiphium*).

Section *Junciformes* include 28 species (Roalson et al., 2021). It is mostly endemic to SA, with its center of diversity in Patagonia (Barros, 1935, 1969; Moore, 1968, 1983), few taxa reaching tropical latitudes through the Andes (Wheeler and Guaglianone, 2003; Jiménez-Mejías and Roalson, 2016) and *C. phalaroides* Kunth entering marginally in Central America (Jiménez-Mejías et al., 2018). In addition, this section is also distributed in SW Pacific (SE Australia, Tasmania, and NZ). It was previously treated as two old different sections mixed in distribution (sects. *Aciculares* G. A. Wheeler and *Junciformes* s.s.) due to apparent morphological differences (Wheeler, 1989). In addition, two species of uncertain phylogenetic position have also been recently ascribed to the current sect. *Junciformes* (*C. phalaroides* and *C. camptoglochin* V. I. Krecz; Jiménez-Mejías et al., 2016a; Roalson et al., 2021). This group is especially interesting from an evolutionary and biogeographical point of view taking into account its disjunction distribution in the Southern Hemisphere, and the fact that it is the second-largest *Carex* section in SA [after sect. *Uncinia* (Pers.) Baill.; Jiménez-Mejías et al., 2018]. Both groups, together with sect. *Fecundae* Kük., constitute the only examples of significantly speciose *Carex* groups (more than 15 species) in SA. The origin of sect. Junciformes has been dated back to the Early Miocene (c. 17 mya; Martín-Bravo et al., 2019), making it also one of the oldest sections of the genus. Species of this group mostly inhabit dry and cold habitats, such as steppe grasslands, fellfields, and rocky outcrops, with fewer species inhabiting mesic and humid environments like bogs, swamps, or forest understories (Barros, 1969; Moore and Edgar, 1970; Wheeler, 1989; Curtis and Morris, 1994).

*Carex* subg. *Psyllophorae* represents an ideal group to study the biogeographic history and evolution of the genus in the Southern Hemisphere, in contrast to its predominant diversification in the Northern Hemisphere. The relatively old origin of the subgenus added to its remarkable distribution pattern makes it an interesting case study to evaluate the role of the different geo-climatic events shaping the diversity of the group, paying special attention to the colonization processes resulting in its currently Gondwanan/Rand-Flora reminiscent disjunct distribution pattern as well as diversification of *Carex* in SA. In particular, we will assess whether the relatively large number of species of sect. *Junciformes* in SA matches any of the types of evolutionary radiation (Simões et al., 2016) and, if so, what triggers could be related to such radiation. Finally, we will try to elucidate the process originating the remarkable disjunction between SA and SW Pacific. To this end, we will perform phylogenetic, biogeographic, and bioclimatic niche evolution analyses.

## Materials and Methods

### Sampling, DNA Amplification, and Alignment

We mainly relied on herbarium materials from 15 herbaria (A, BR, CHR, CONC, GOET, E, M, MA, MO, MSB, NY, SI, UPOS, and WS; codes according to Thiers, 2020) to study 45 species (49 taxa) belonging to subg. *Psyllophorae* (Supplementary Table 1): 22 species plus four varieties from sect. *Junciformes* (all except *C. archeri* Boott, *C. boelckeiana* Barros, and *C. moorei* G. A. Wheeler); all 7 species of sect. *Psyllophorae*; and 17 included in sect. *Schoenoxiphium* (all except *C. acocksii* C. Archer, *C. chermezonii* Luceño and Martín-Bravo, *C. gordon-grayae* Luceño, Márq.-Corro and Sánchez-Villegas, and *C. sciocapensis* Luceño, Márq.-Corro and Sánchez-Villegas; see Luceño et al., 2021 for more information). Our dataset comprises 87% of all the extant diversity known for subg. *Psyllophorae* (following Roalson et al., 2021 with some modifications; 53 species) including all main lineages and representing the geographical and morphological variability of the subgenus. *Carex phalaroides* s.l. constitutes a complex species much in need of detailed study; so we opted for a synthetic view and considered it as a single species (herein *C. gibertii* G. A. Wheeler, *C. moesta* Kunth, and *C. hypsipedos* C. B. Clarke are treated as *C. phalaroides*). We used as outgroup one or two representatives of each of the other five *Carex* subgenera (Villaverde et al., 2020): *C. hypolytroides* Ridl. and *C. siderosticta* Hance (subg. *Siderostictae* Waterway), *C. canescens* L. and *C. gibba* Wahlenb. (subg. *Vignea*), *C. flava* L. and *C. dissitiflora* Franch. (subg. *Carex*), *C. arctogena* Harry Sm. (subg. *Euthyceras* Peterm.), and *C. meridensis* (Steyerm.) J. R. Starr (subg. *Uncinia* Pers.).

We used four DNA regions, the nrDNA ITS and ETS, and the ptDNA *mat*K and *rps*16. These markers were selected because they have been used successfully in previous studies in *Carex* in general and in subg. *Psyllophorae* in particular (Gehrke et al., 2010; Jiménez-Mejías et al., 2016a; Villaverde et al., 2017b; Márquez-Corro et al., 2020). DNA extraction and sequence amplification followed Jiménez-Mejías et al. (2016a), except for *rps16* amplification that was performed as indicated in Shaw et al. (2005). All PCR products were sequenced by Macrogen (Madrid, Spain). Sequence chromatograms were manually edited using Geneious v. 11.0.2 (Biomatters Ltd., Auckland, New Zealand).

Four independent matrices were compiled, each one containing sequences for one of the DNA regions (ITS, ETS, *mat*K, or *rps*16). The sequences were automatically aligned with Muscle v.3.8.425 (Edgar, 2004) and alignments were manually corrected in the flanking regions of indels. Informative indels were coded as a binary character using SeqState v.1.4.1. according to Simmons and Ochoterena (2000) simple coding method. A complete multiaccession matrix (105 accessions, 23.26% missing data) was built concatenating all markers.

### Phylogenetic Dating and Diversification Rate Analyses

We performed maximum likelihood (ML) and Bayesian Inference (BI) phylogenetic analyses using RAxML v.8.2 (Stamatakis, 2014) and MrBayes v.3.2 (Ronquist et al., 2012), as implemented in CIPRES Science Gateway (Miller et al., 2010), with parameters detailed in Supplementary Data 1.

We inferred divergence times in subg. *Psyllophorae* with a Bayesian analysis implemented in BEAST v.1.10 (Suchard et al., 2018) establishing three primary calibration points based on reliable *Carex* fossils (Jiménez-Mejías et al., 2016b): *C. colwellensis* (Eocene: 38.0-33.9 mya) for the crown node of the genus, *C. marchica* (Early Miocene: 23.0-16.0 mya) and *C. hartauensis* (late Oligocene: 28.1-23 mya) for the stem node of subg. *Vignea* and *Carex*, respectively (Table 2). We also used a secondary calibration point for the crown node of subg. *Psyllophora* (24.41 mya; Martín-Bravo et al., 2019). Prior node age distributions and analyses settings are detailed in Supplementary Data 1.

**Table 2:** Three calibration points based on fossils and a secondary one were used to date the phylogenetic tree of *Carex* subg. *Psyllophorae* using BEAST.

**Calibration points**	**Placement**	**Age (mya)**	**BEAST node parameters**	**References**
*C. colwellensis*	(A) Crown node of genus *Carex*	Eocene (Priabonian; 38.0-33.9 mya)	Normal distribution (mean: 35.95 mya; stdev: 1.2)	Jiménez-Mejías et al., 2016b
*C. marchica*	(B) Stem node of *Carex* subg. *Vignea*	Early Miocene; (23.0-16.0 mya)	Lognormal distribution (offset: 16.0; mu: 0.4; sigma: 1.0)	Jiménez-Mejías et al., 2016b
*C. hartauensis*	(C) Stem node of *Carex* subg. *Carex*	Late Oligocene (Chattian; 28.1-23.0 mya)	Lognormal distribution (offset: 23.0; mu: 0.1; sigma: 1.0)	Jiménez-Mejías et al., 2016b
Secondary calibration	(D) Crown node of *Carex s*ubg. *Psyllophorae*	Late Oligocene (Chattian; 24.41 mya in Martín-Bravo et al., 2019)	Normal distribution (mean: 22.17 mya; stdev: 1.0)	Martín-Bravo et al., 2019

Diversification rates for subg. *Psyllophorae* were estimated on the dated singleton tree using the speciation-extinction model implemented in BAMM (BAMMtools package; Rabosky et al., 2014) using R v.3.6 (R Development Core Team, 2019). This analysis was performed in order to estimate the diversification rates by the effective sample size of the log-likelihood and number of shift events along branches using reversible jump Markov chain Monte Carlo (rjMCMC) to infer sample mixtures of distinct evolutionary rate dynamics across the branches. Four chains of 1.000.000 generations, saving trees each 1,000 (see Supplementary Data 1 for more information) were performed.

### Bioclimatic Niche Analyses

Occurrences belonging to sect. *Junciformes* (Supplementary Table 2) were downloaded from GBIF (https://www.gbif.org/), iDiGBio (https://www.idigbio.org), and the ALA database (https://www.ala.org.au/). In addition, we manually georeferenced 154 vouchers from 14 herbaria (A, CONC, E, LIL, M, MICH, MO, MSB, NY, SI, TRIER, UPOS, US, and WS), as well as 115 records obtained from literature sources (Barros, 1948, 1950, 1957; Wheeler, 1988, 1989, 1998; Wheeler and Muñoz-Schick, 1990; Wheeler and Guaglianone, 2003; Wheeler and Beck, 2011; Jiménez-Mejías et al., 2020). We cleaned the resulting database by removing duplicates and unreliable records after visual inspection, and finally, 616 occurrences were recovered (Supplementary Table 2).

We retrieved 37 bioclimatic and ecophysiological variables from WorldClim2 (Fick and Hijmans, 2017) and Envirem (Title and Bemmels, 2018) to characterize the bioclimatic niche of our study group. Our variable selection procedure (Supplementary Data 1) retained five bioclimatic variables that contributed more than the rest to the variability of our data, and have easy biological interpretation for our species: Annual mean temperature (AMT), temperature annual range (TAR), mean temperature of the driest quarter (MTDQ), annual precipitation (AP), and terrain roughness index (TRI). These variables illustrate species-climatic limits regarding water availability (AP), temperature optimum (AMT), and limits (TAR, MTDQ), as well as microclimatic determinants driven by topography (TRI). We conducted a principal component analysis (PCA) of the retained variables using the package ggplot2 (Wickham, 2006) and the function *prcomp* in R for visualizing the environmental space of sect. *Junciformes*.

**Figure 2:**
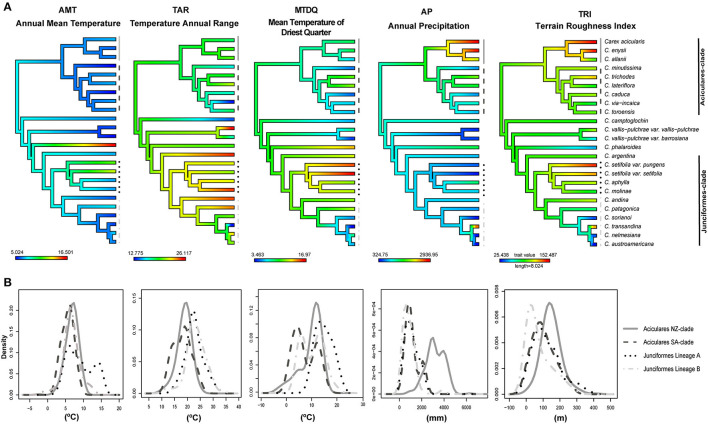
**(A)** Bioclimatic niche reconstruction at ancestral nodes for five continuous environmental variables of the calibrated tree for the *Carex* sect. *Junciformes*. The ML reconstruction is represented as gradational colors along the branches. Higher values are displayed in red, intermediate in green, and low in blue. **(B)** Comparison of density plots expressing the frequency of distribution for the same uncorrelated variables in the four clades belonging to *Carex* sect. *Junciformes*. These clades are also represented in Figure 3C to the right of the phylogeny. ML, maximum likelihood.

We performed ancestral state reconstruction of ecological preferences for sect. *Junciformes* using the dated pruned singleton tree and the mean value for each retained variable in each species as implemented in the package phytools in R (Revell, 2012; Figure 2A). We previously assessed two models of continuous trait evolution [Brownian motion (BM) and Ornstein-Uhlenbeck (OU)] and selected the best using corrected Akaike Information Criterion (AICc) (fitContinuous function in geiger package; Harmon et al., 2008).

In order to study fine-level ecological differences within sect. *Junciformes*, we obtained and plotted response curves for each retained variable for the main sister lineages (Figure 2B) and single species (Supplementary Figures 1, 2). To characterize the environmental space for a whole lineage, we merged all occurrences of the corresponding species. To further evaluate the Phylogenetic Niche Conservatism hypothesis between sister lineages from SA and NZ within the Aciculares-clade (see Results), we also performed tests of similarity and equivalence (Warren et al., 2008; Broennimann et al., 2012) implemented in the package ecospat (Di Cola et al., 2017). We also measured the niche overlap between these sister clades with Schoener's D index (Schoener, 1968).

### Biogeographic Analyses

**Figure 3:**
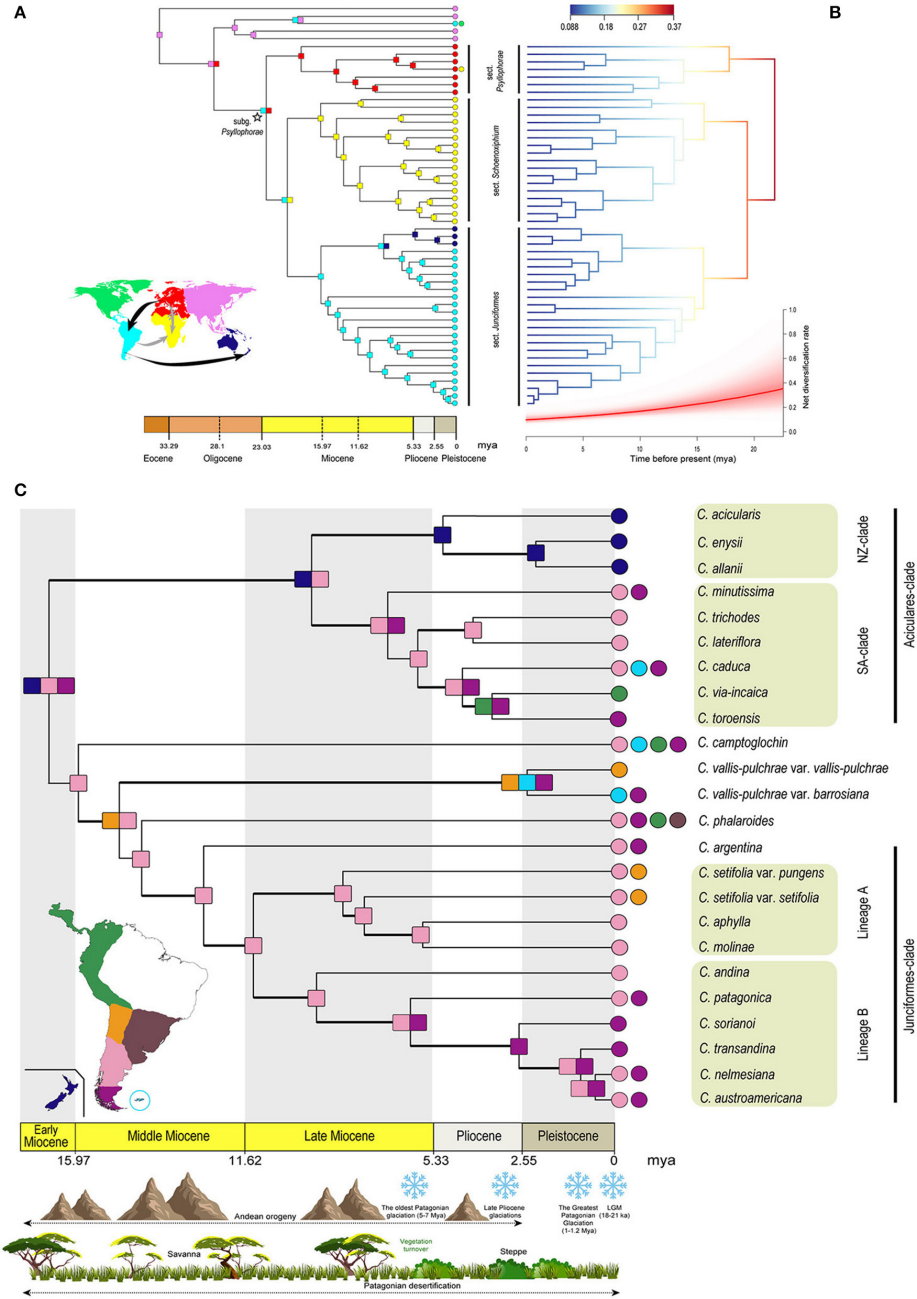
**(A)** Chronogram based on the dated phylogeny of *Carex* subg. *Psyllophorae*. Square nodes and circle terminals with different colors represent the most probable area or combination of areas inferred by the DIVA-like model in the AAR using BioGeoBEARS. Colors are according to the world map representing the regions coded for the biogeographic analysis. Arrows in the map show the direction of migration among the different regions inferred by the AAR. Arrows in black color display a clear colonization route inferred by the AAR, while ones in gray color display uncertainty. Two crossed lines on the gray arrow from Western Palearctic to the Afrotropical region display an uncertain dispersal or vicariance pattern between both the continents. **(B)** Phylorate plot from the analysis of diversification rate using BAMM, based on the dated phylogeny of *Carex* subg. *Psyllophorae* (excluding outgroup). Tree branch color indicates the model-averaged net diversification rates along the branches. Below is represented the net diversification rate through time with a red line displaying the mean and the shaded range of its rjMCMC confidence interval. **(C)** Chronogram based on the dated phylogeny including only *Carex* sect. *Junciformes*. Square nodes and circle terminals with different colors represent the most probable area or combination of areas inferred by the DEC model in AAR specifically performed for SA and NZ. Colors are according to the map representing the South American and NZ regions coded for the biogeographic analysis. The main historical geo-climatic events since the origin of this group are shown with representative drawings in the timeline. Bold branches represent well-supported nodes (PP > 0.9). DIVA, dispersal-vicariance analysis; AAR, ancestral area reconstruction; BAMM, Bayesian Analysis of Macroevolutionary Mixtures; rjMCMC, reversible jump Markov chain Monte Carlo; DEC, dispersal-extinction cladogenesis; SA, South America; NZ, New Zealand.

Ancestral area reconstruction (AAR) was performed using the package BioGeoBEARS (Matzke, 2014a) in R. We performed two different biogeographic reconstructions focusing on two different evolutionary-geographic scales. First, we performed a large-scale analysis based on our complete sampling for the whole subg. *Psyllophorae*, coding species distribution according to six biogeographical regions (Figure 3A): North America, SA, Eastern Palearctic, Western Palearctic, Afrotropical region, and Australasia (including NZ). For species in subg. *Psyllophorae*, we obtained distributions from the World Checklist of Selected Plant Families (WCSP) (Govaerts et al., 2020), while for each outgroup tip, the coded distribution was obtained from the ancestral area of the correspondent subgenus as inferred by Martín-Bravo et al. (2019). Second, we focused only on sect. *Junciformes* to explore in detail its biogeography, delimiting subregions within the Neotropics and NZ, based on their current (Supplementary Figure 3) and inferred present potential distribution (Supplementary Figure 4; see Supplementary Data 1 for methodological details). As a result, we considered seven areas (Figure 3C): Northern Andes, Central Andes, Northern Patagonia (N Patagonia), S Patagonia, Atlantic South America, Falklands, and NZ. As input data, we used the pruned calibrated tree and a file recording the presence/absence of species in each area.

Both datasets were analyzed under three different models: Dispersal-Extinction-Cladogenesis (DEC; Ree and Smith, 2008), a likelihood version of the Dispersal-Vicariance analysis (DIVA-like; Ronquist, 1997), and a likelihood interpretation of BayArea model (Landis et al., 2013) implemented in BioGeoBEARS (BAYAREA-like; Matzke, 2014a), and each of these in combination with the founder-event speciation (+J model; Matzke, 2014b). The fit of the different models was tested using AICc (Burnham and Anderson, 2002).

## Results

### Phylogenetic, Divergence Time and Diversification Analyses

Subgenus *Psyllophorae* was recovered in a strongly supported monophyletic group (Supplementary Figure 5) including sects. *Psyllophorae, Schoenoxiphium*, and *Junciformes*, which also formed well-supported clades. Section *Psyllophorae* (Clade A) was sister to Clade B which in turn contained sects. *Junciformes* and *Schoenoxiphium* as sister groups (see Supplementary Data 2 for a detailed description of phylogenetic results).

Divergence times obtained with BEAST are shown in Supplementary Figure 6. The crown age of subg. *Psyllophorae* coincided with the early Miocene (mean = 22.55 mya, 95% highest probability density (HPD) = 20.76–24.44 mya). The most recent common ancestors (MRCA) of sect. *Psyllophorae* (Clade A; mean = 18.40 mya, 95% HPD = 15.01-21.56 mya) and sects. *Schoenoxiphium* and *Junciformes* (crown node of Clade B; mean = 20.04 mya, 95% HPD = 17.25-22.84 mya) were inferred to have diversified just after the subgenus as a whole, still in the early Miocene. Interestingly, both sections from the Southern Hemisphere were originated almost at the same time in the early Miocene (Clade B1: sect. *Schoenoxiphium*, mean = 16.10 mya, 95% HPD = 12.5-19.47, and Clade B2: sect. *Junciformes*, mean = 16.05 mya, 95% HPD = 12.86-19.17 mya). Within sect. *Junciformes*, we found two main clades diversified in late Miocene (Aciculares-clade, mean = 8.60 mya, 95% HPD = 5.6–11.89 mya; core Junciformes, mean = 10.25 mya, 95% HPD = 7.89–12.82 mya). On the one hand, Junciformes-clade (including the monospecific lineage of *C. argentina*) seems to have diversified during the middle Miocene (mean = 11.66 mya, 95% HPD = 9.04–14.38 mya). The MRCA of core Junciformes was dated to the late Miocene (mean = 10.25 mya, 95% HPD = 7.89–12.82 mya), and in turn, the Lineages A and B derived from this ancestor were also inferred to have differentiated in the late Miocene (Lineage A: mean = 7.71 mya, 95% HPD = 5.6-10 mya; Lineage B: mean = 8.46 mya, 95% HPD = 6.14-10.9 mya). On the other hand, within Aciculares-clade, the diversification of SA-clade was during the late Miocene (mean = 6.44 mya, 95% HPD = 4.06–8.95 mya), whereas NZ-clade probably occurred a bit later during early Pliocene (mean = 4.87 mya, 95% HPD = 2.79–7.28 mya).

Bayesian Analysis of Macroevolutionary Mixtures (BAMM) analyses estimated a similar and constant diversification pattern along the three sections (*Psyllophorae, Schoenoxiphium*, and *Junciformes*; Figure 3B) of subg. *Psyllophorae*. In addition, it did not detect significant shifts in diversification rate.

### Bioclimatic Niche Analyses

The PCA (Supplementary Figure 7) of the variables using mean values for all occurrences per species of sect. *Junciformes* displayed a clear trend toward variables related to temperature along the first principal component, which explained 42.1% of the total variance. Precipitation (AP) and terrain roughness (TRI) were negatively correlated with this axis and displayed lower values than temperature ones, while they contributed positively to the second principal component that showed 32.4% of the total variance explained. Therefore, the cumulative proportion of the variance explained by both principal components was 74.5%.

The best evolutionary model for bioclimatic reconstruction over sect. *Junciformes* phylogenetic tree according to the corrected AICc value was the Ornstein–Uhlenbeck model (BM = 112.10, log-likelihood = −53.76; OU = −34.99, log-likelihood = 20.76). The bioclimatic niche evolution of the five selected variables on the obtained phylogeny is shown in Figure 2A, as well as density functions exhibiting the frequency of the distribution of the bioclimatic variables in Figure 2B. In general, similar values across internal nodes of the phylogeny were retrieved for each independent variable (Figure 2A). This is also depicted by the mostly overlapping and relatively narrow peaks for the different sister clades (Aciculares-SA vs. NZ; Junciformes lineages A vs. B; see also Supplementary Figures 1, 2) in the response curves (Figure 2B). The deep nodes along the phylogeny displayed medium values on their bioclimatic preferences, while tips revealed the existence of more extreme values (Figure 2A).

Despite the general scenario of relatively uniform bioclimatic values across sect. *Junciformes*, some degree of heterogeneity was observed for certain variables in particular lineages. Thus, TAR was the variable that showed the highest environmental heterogeneity along the phylogeny, with contrasting bioclimatic preferences for Junciformes-clade (*C. andina Phil., C. molinae Phil., C. patagonica* Speg., and *C. setifolia* Kunze). The monospecific lineage of *C. phalaroides* showed high values for AMT in comparison to low-medium values in the rest of the species of sect. *Junciformes*. In addition, closely related species of Aciculares NZ-clade presented contrasting bioclimatic preferences across different variables, such as AP and TRI, as well as TAR and TRI for some species belonging to Junciformes-clade (*C. setifolia* and *C. transandina*). Finally, the high values displayed in the irregularity of terrain (TRI) were associated with species that inhabit a wider altitude range across NZ and SA. In addition, *C. setifolia* revealed higher values of MTDQ in comparison to medium-lower ones for the other tips. Otherwise, *C. transandina* G. A. Wheeler displayed contrasting values for AP and TRI, more similar to species from Aciculares NZ-clade than Junciformes Lineage B to which it is phylogenetically more related (Figure 2A and Supplementary Figure 2).

The PCA comparing the e-space for NZ and SA clades (Aciculares-clade) revealed a large overlap between both groups within both principal components (67% explained of the total variance; Supplementary Figure 8). This niche overlapping was also revealed by Schoener's D index (D = 0.523). In addition, pairwise statistical comparison for similarity and equivalence tests suggested that these clades were also significantly more similar and equivalent than expected by chance (*p* < 0.01).

### Biogeographic Analyses

Dispersal-vicariance analysis (DIVA-like model) yielded lower corrected AICc values than DEC and BAYAREA-like model (76.84, 81.15, and 111.8, respectively), with ΔAICc > 2 (Burnham and Anderson, 2002). DIVA-like and DEC models resulted in biologically congruent reconstructions. However, the BAYAREA-like model displayed scarce uncertainty for node inferences although this model is more limited in the number of evolutionary scenarios considered (i.e., vicariance is not considered). In order to simplify the results, we mainly focused on the DIVA-like model (Figure 3A). Results from DEC and BAYAREA-like models are presented in Supplementary Figures 9, 10, respectively. The stem node of subg. *Psyllophorae* (Oligocene) was restricted to the Palearctic, from where it dispersed to SA (crown node widespread in the Western Palearctic and SA, early Miocene). The differentiation of sect. *Psyllophorae* took place in the Western Palearctic from the early Miocene. For the clade embracing sects. *Schoenoxiphium* and *Junciformes*, the ancestor was recovered as widespread in the Afrotropical region and SA during the early Miocene. The differentiation of each of these two sections implied the constriction of their crown node to one of the two landmasses. While the arrival to SA necessarily implied a LDD event from the Old World, the early Miocene colonization of the Afrotropical region remains obscure, since we cannot rule out the dispersal from SA or dispersal/vicariance from the Western Palearctic. Finally, a LDD event to SW Pacific from SA across the Mio-Pliocene boundary led to the more recent diversification of the NZ-clade (Figure 3A). Within-area lineage diversification from the middle Miocene onwards was inferred for the remaining subclades within the three sections commented above (Figure 3A and Supplementary Figures 9, 10).

Regarding the biogeographic reconstruction for sect. *Junciformes*, the DEC model yielded lower AICc values than the DIVA-like and BAYAREA-like models (141.2, 142.4, and 143.3, respectively), although with ΔAICc <2 between DEC and DIVA-like models. Thus, we decided to focus on the DEC model (Figure 3C). Results for DIVA-like and BAYAREA-like models are presented in Supplementary Figures 11, 12, respectively. A widespread ancestral area including Patagonia as a whole and NZ was inferred for the crown node of sect. *Junciformes* at the late early Miocene, although uncertainty was present under the three biogeographic models tested with ranges probabilities below 0.10 (Figure 3C and Supplementary Figures 11, 12). DIVA-like model inferred N Patagonia, while BAYAREA-like model inferred both N-S Patagonia and Central Andes.

In the Aciculares-clade and the DEC model, a widespread area in N Patagonia and NZ was inferred for the MRCA (late Miocene). On the one hand, a LDD event from N Patagonia gave rise to the NZ-clade at the Mio-Pliocene boundary. On the other hand, from the late Miocene onwards, the diversification of SA-clade took place within N-S Patagonia, with at least two later Plio-Pleistocene independent range expansions (to the Falklands *-C. caduca* Boott- and Northern Andes *-C. via-incaica* Jim. Mejías and Roalson-). Remarkably, a similar scenario of Patagonian diversification was inferred for the Junciformes-clade, although starting earlier (from the Middle Miocene) than in the Aciculares-clade, and restricted to the N Patagonia. Subsequent expansions took place southwards to the S Patagonia (Pleistocene; Lineage B), and northwards to the Central Andes (from the Late Miocene; *C. setifolia*). The isolated lineages of *C. camptoglochin, C. vallis-pulchrae*, and *C. phalaroides* underwent unprecedented range expansions within the section, including multiple extra-Patagonian regions.

We preferred models not accounting for the J parameter for the AAR analyses as these results should be treated with caution (Ree and Sanmartín, 2018).

## Discussion

### Systematic Implications for Subg. *Psyllophorae*

Our sanger-based phylogenetic findings suggest that subg. *Psyllophorae*, as delimited by Roalson et al. (2021), is a well-supported monophyletic group (Supplementary Figure 5), but not including *C. baldensis* L. and *C. curvula* All. The placement of these two species in this subgenus needs further study and it was considered provisional by Roalson et al. (2021) as they were placed on a very short branch in the phylogenomic reconstructions (Villaverde et al., 2020). Three well-supported major clades are identified within subg. *Psyllophorae*, each one representing a section (*Psyllophorae, Schoenoxiphium*, and *Junciformes*), unlike previous phylogenies where lower taxonomic and molecular sampling yielded less robust reconstructions (Jiménez-Mejías et al., 2016a; Martín-Bravo et al., 2019). The prolonged isolated evolution of some species (Supplementary Figure 6) has yielded a remarkable morphological differentiation within sections (e.g., *C. distachya, C. camptoglochin*, and *C. phalaroides*), which has traditionally caused taxonomic problems in their sectional delimitation (Wheeler and Guaglianone, 2003; Luceño, 2008; Silveira and Longui-Wagner, 2012). The monophyly of sect. *Schoenoxiphium* is well-supported (Clade B1; Supplementary Figure 5), which agrees with previous studies focusing on that group (Luceño et al., 2021, and references therein) despite resolution problems reported for its inner phylogenetic structure. Meanwhile, *C. camptoglochin* and *C. phalaroides* are nested within sect. *Junciformes* clade, supporting their treatment within that section (Roalson et al., 2021). Section *Aciculares* as conceived by Wheeler (1989) is not monophyletic due to the position of *C. transandina* and *C. vallis-pulchrae* with respect to the members of sect. *Aciculares* (Supplementary Figure 5). As currently circumscribed, sect. *Junciformes* is primarily characterized by the presence of inflorescences formed by a single androgynous spike, usually dense, and with a staminate tip concealed by the female part. The only exception is *C. phalaroides*, with multispicate inflorescences, which seems a reversion to the ancestral state of the genus (Roalson et al., 2021). In addition, *C. camptoglochin*, which displays reflexed utricles with a protruding rachilla that may play an epizoochorous role (Villaverde et al., 2017a), has been traditionally included in sect. *Leucoglochin* Dumort. primarily because of this distinctive morphological character (Wheeler and Guaglianone, 2003).

### Large-Scale Biogeography in Subg. *Psyllophorae*: Rand-Flora vs. Gondwanan Patterns

Subgenus *Psyllophorae* is inferred as one of the oldest lineages of *Carex*, dated to the early Miocene (22.55 mya), with previous studies retrieving even slightly older ages around the Mio-Oligocene boundary (24.4 mya; Martín-Bravo et al., 2019). In congruence with the origin and early diversification of *Carex* in the Eastern Palearctic (Martín-Bravo et al., 2019), our results point to an ancient migration from the Northern to the Southern Hemisphere, specifically from the Palearctic to SA (Figure 3A). Thus, although the biogeographic reconstruction inferred the Western Palearctic and SA continent as the ambiguous ancestral area for the crown node of subg. *Psyllophorae*, the immediate parent node was inferred to be exclusively distributed in the Northern Hemisphere (Eastern Palearctic or Western Palearctic). In a previous reconstruction using a representative sampling for the whole genus, the Western Palearctic was revealed as the origin of subg. *Psyllophorae* (Martín-Bravo et al., 2019).

Allopatric differentiation is suggested for the three main sectional lineages of subg. *Psyllophorae* originating in different regions (sect. *Psyllophorae* in the Western Palearctic, sect. *Schoenoxiphium* in the Afrotropical region, and sect. *Junciformes* in SA) and diversifying almost exclusively within each one (except sect. *Junciformes*, which also colonizes the SW Pacific for more than 15 million years (Figure 3A). A remarkably synchronous timing of the origin was inferred for these three main lineages (16–18 mya).

As expected, a primary (tectonic) Gondwanan disjunction has to be ruled out for the Southern Hemisphere lineages (sects. *Schoenoxiphium* and *Junciformes*) because their ages long postdate the split of the southern supercontinent (c. 16 mya vs. 135–105 mya; McLoughlin, 2001), unlike what is found in other, much older groups of plants (Araucariaceae, Biffin et al., 2010; *Dicksonia*, Noben et al., 2017). It is unclear whether the Afrotropical region was colonized from SA by LDD or from the Western Palearctic (Figure 3A). In the latter case, since this colonization occurred in the early Miocene (c. 20 mya) the disjunction is probably too old to fit a climate-driven Rand-Flora vicariance (Mairal et al., 2017), while LDD may not be ruled out (Míguez et al., 2017). Moreover, the origin of sect. *Schoenoxiphium* seems to be in central Southern Africa, which also dismisses the Rand-Flora pattern (Márquez-Corro et al., 2020). An additional, more recent (probably Pleistocene) colonization of the Afrotropical region from the Western Palearctic by LDD can be identified within sect. *Psyllophora* involving *C. peregrina* Link (Figure 3A). Remarkably, this constitutes the only recent out-of-continent dispersal within the subgenus.

### Phylogeography of Sect. *Junciformes*: Diversification in South America and Colonization of New Zealand

The diversification of sect. *Junciformes* within SA started close to their origin in the middle Miocene and was characterized by relatively constant cladogenesis (especially in the Junciformes-clade), while diversification rate decreased over time (Figures 3B,C). The group remained confined and diversified in N Patagonia for several million years (Figure 3C). This long-permanence and within-area diversification is known in other groups at comparative evolutionary levels (Mathiasen and Premoli, 2010; Otero et al., 2019; Benítez-Benítez et al., 2021). The persistence of the group in N Patagonia and lack of evidence of early presence at lower latitudes suggest that sect. *Junciformes* colonized the Southern Cone from the Northern Hemisphere by direct LDD from the Western Palearctic (Figures 3A,C). Contrastingly, most cold-adapted Neotropical plant groups used the American cordillera as a corridor into SA (Zemlak et al., 2008; Xu et al., 2009; He and Sun, 2017). It would not be until the latest stages of the orogeny that the Andes served as a natural passageway for multiple colonizations in sect. *Junciformes*, including northward migrations from Patagonia (*C. camptoglochin, C. setifolia, C. vallis-pulchrae*, and *C. via-incaica*). This could have been favored by the concurrent, Pliocene cooling of the low-latitude Andes (Roberts et al., 2017). Both, the early permanence of sect. *Junciformes* in N Patagonia and the posterior colonizations within SA entailed no strong ecological innovations but a relative niche conservatism (Figures 2, 3C). Our bioclimatic reconstructions suggest that such ecological conservation is favored by selection (Figure 2A, OU model; Blomberg et al., 2020). Accordingly, the changing environment in SA (Rabassa, 2008) instead of inducing niche shifts in sect. *Junciformes* entailed the range expansion as similar suitable niches became available. By contrast the entrance of *C. phalaroides* in multiple areas out of Patagonia implied striking changes in bioclimatic preferences, especially regarding AMT (Figure 2A). This is expectable since this species inhabits radically different, much more temperate habitats (Atlantic Forest, Pampas, montane tropical forest) in comparison to the other species belonging to sect. *Junciformes*.

In the late Pliocene and during the Pleistocene, several groups (Junciformes-clade Lineage B and Aciculares-clade SA-clade) underwent *in situ* diversification either in N or S Patagonia. In addition, these clades present changes in bioclimatic preferences at very shallow levels, especially in the Junciformes-clade Lineage B (Figure 2). These could be interpreted as a result of local adaptation in glacial refugia, as reported in other plant genera in different regions around the world (Tremetsberger et al., 2009; Premoli et al., 2010; Kremer, 2016), as well as in other *Carex* groups (Benítez-Benítez et al., 2018). Many plant groups were able to survive Patagonian glaciations in high latitude refugia (see references in Table 1) since ice sheets were generally not extensive after the Greatest Patagonian Glaciation (1–1.2 mya; Rabassa, 2008). During the Pleistocene, major Patagonian rivers have been invoked as barriers for allopatric speciation in plants (Jakob et al., 2009; Sede et al., 2012; Cosacov et al., 2013), but these do not seem to have acted as barriers in *Carex* given its striking capacity for LDD (Villaverde et al., 2017a). In other plant groups, glacial survival in Patagonia seems to be conditioned by the aridification and establishment of the steppe (Jakob et al., 2009; Cosacov et al., 2010; Sede et al., 2012). In sect. *Junciformes*, the steppe may have played a certain role as a barrier since these species hardly penetrate this biome (Supplementary Figure 3).

Section *Junciformes* colonized SW Pacific (NZ and probably also SE Australia and Tasmania) from SA (Aciculares-NZ clade; Figure 3A), a biogeographic pattern also reported in other families of plants (Von Hagen and Kadereit, 2001; Meudt and Simpson, 2006; Otero et al., 2019). Whether the colonization took place by direct LDD or stepping stone is unclear, despite the absence of sect. *Junciformes* in circum-Antarctic archipelagos seem to point to a direct LDD as the most plausible explanation. In any case, inferred divergence ages (late Miocene-Pliocene; Supplementary Figure 6) also rule out the role of Antarctica as the last tundra remnants of this region are reported to disappear around the Middle Miocene (Lewis et al., 2008; but see Barrett, 2013). New Zealand species inhabit alpine habitats which became available with the orogeny of the Southern Alps starting in the Pliocene (Heenan and McGlone, 2013), which mostly matches the crown node age inferred for this group (Figure 3C and Supplementary Figure 6). Interestingly, the colonization of NZ entailed a remarkable long-term niche conservatism within Aciculares-clade for different variables (AMT, TAR, MTDQ; Figure 2), which indicates that the lineage was somehow preadapted to the newly colonized habitats. Nonetheless, a certain degree of local adaptation is revealed by the somewhat different values for AP and TRI (Figure 2).

### Diversification Patterns in Subg. *Psyllophorae* and Their Relation to Niche Evolution

The phylogenetic reconstruction of subg. *Psyllophorae* shows relatively synchronous cladogenesis through time and an unbalanced topology, with a relatively poor lineage (sect. *Psyllophorae*) sister to a much more diversified group (sects. *Schoenoxiphium*-*Junciformes*; Figure 3B). This has sometimes resulted in a ladderized rather than bifurcating topology (Crisp and Cook, 2005; Vargas and Zardoya, 2014), with frequently long branches and deep nodes originating old monotypic and/or species-poor lineages. Their stem nodes date as old as the early-middle Miocene. Interestingly, this pattern has been found in the three sections: *Psyllophorae* (*C. distachya* Desf., c. 18 mya; *C. illegitima* Ces., 12 mya); *Schoenoxiphium* (*C. multispiculata* Luceño and Martín-Bravo - *C. lancea* (Thunb.) Baill., c. 11 mya), and *Junciformes* (*C. camptoglochin*, c. 15 mya; *C. vallis-pulchrae*, c. 14 mya; *C. phalaroides*, c. 14 mya).

Diversification rates seem to progressively decrease in subg. *Psyllophorae* (Figure 3B), which agrees with the previous findings in Martín-Bravo et al. (2019) using a larger sampling of *Carex* species but a smaller set of sect. *Junciformes* taxa. As stated before, sect. *Junciformes* is one of the only three Neotropical *Carex* groups with more than 15 species (see Introduction). This lack of significant increase in diversification rates points to a process of disparification rather than true evolutionary radiation (Simões et al., 2016). Thus, its current relatively high diversity would be simply the result of the progressive accumulation of changes during its long evolutionary history.

The allopatric distribution of the three sections in different landmasses (Figure 1) could have imposed differential intrinsic/extrinsic factors influencing their diversification. The limited ecological diversification revealed by our bioclimatic niche analysis in sect. *Junciformes* (Figure 2B) provides insights into the intrinsic conditionants of its evolution. A relatively conserved niche in this lineage, perhaps constrained by its own ecological limits, may have prevented a significant increase in diversification rates. It means that the ecologically suitable space could have been early filled. In particular, the case of the NZ-clade is remarkable, since the group hardly diversified after colonizing NZ, also involving limited ecological (Figure 2) and morphological change (Hamlin, 1962; Edgar, 1970), which strongly contrasts with other spectacular cases of morphological/ecological adaptive radiations within NZ (Wagstaff et al., 2002; Glenny, 2004; Meudt and Simpson, 2006). Furthermore, other *Carex* groups (sects. *Spirostachyae* and *Uncinia*; Roalson et al., 2021) underwent remarkable radiations in NZ, but they could have arrived earlier to the archipelago than NZ-clade of sect. *Junciformes* (Martín-Bravo et al., 2019), perhaps preventing the establishment of its species through high density blocking (Slingsby and Verboom, 2006; Pender et al., 2021).

Further studies are needed to characterize in detail the niche of the other sections of subg. *Psyllophorae*. On the one hand, sect. *Schoenoxiphium* was inferred to have originated in the Drakensberg range in South Africa, where its current center of diversity is still located (Márquez-Corro et al., 2020). In this area, species distributions are often overlapping, but with frequent turnover along various ecological gradients (elevation, wetness, forest to grassland; Luceño et al., 2021). Thus, speciation in this lineage appears to have been sympatric and driven by ecological factors. On the other hand, sect. *Psyllophorae* combines widely distributed species (*C. distachya* and *C. pulicaris*) which are partly sympatric with several allopatric narrowly distributed species (Jiménez-Mejías and Luceño, 2011). Interestingly, while it is the poorest diversified section within subg. *Psyllophorae*, its species display remarkably different ecological preferences, ranging from Mediterranean shrublands to mountain bogs (Borges et al., 2008; Luceño, 2008; Silva et al., 2010; Verdcourt, 2010; Gehrke, 2011).

## Conclusion

The present study provides new insights into the biogeographic and diversification patterns of the Southern Hemisphere and in particular Patagonia, one of the least studied areas of SA. It also sheds light on the phylogenetic structure of one of the oldest lineages of *Carex* (subg. *Psyllophorae*, dated to the early Miocene), which early diversified allopatrically in three different continents: sect. *Psyllophorae* in Western Palearctic, sect. *Schoenoxiphium* in Afrotropical region, and sect. *Junciformes* in SA. In particular, the early diversification of the latter section involved the differentiation of a group of species mostly restricted to a single landmass (SA), with its diversity centre in N Patagonia and a single direct LDD to SW Pacific. This trans-Pacific disjunction entails a striking niche conservatism, which implies that these species seem to have been preadapted to ecological requirements made available around the time of arrival, but probably limited subsequent *in situ* diversification. Later diversification in Patagonia pointed to an important role of Plio-Pleistocene glaciations and the triggering of multiple colonizations toward other SA regions. Furthermore, the Andes acted as a corridor toward the north, an inverse pattern to that reported for the colonization of most Northern Hemisphere cold-adapted plants into SA. The section as a whole seems to have developed geographic speciation with slight ecological differentiation as bioclimatic variables show relatively homogeneous values among species of the same lineage/clade. However, we cannot rule out certain local adaptations for the group at the microevolutionary level.

## Data Availability Statement

The original contributions presented in the study are included in the article/Supplementary Materials, further inquiries can be directed to the corresponding author/s.

## Author Contributions

CB-B carried out the laboratory work, performed the analyses, and drafted the manuscript. PJ-M and SM-B conceived the idea, collected plant material, and drafted the manuscript. AO carried out biogeographic analyses. PG-M collected occurrences data for bioclimatic niche evolution analyses. ML, KF, and SD collected plant material. All authors contributed to the writing of the final version.

## Funding

This support was carried out with financial support by the Spanish Ministry of Science and Innovation (project PID2020-113897GB-I00) and the Regional Government of Madrid, Spain (Macondo SI1/PIJ/2019-00333). CB-B was supported by a Predoctoral Fellowship Program grant (FPU16/01257) from the Spanish Ministry of Universities, and SM-B by a José Castillejo grant (CAS19/00253), from the Spanish Ministry of Science and Innovation.

## Conflict of Interest

The authors declare that the research was conducted in the absence of any commercial or financial relationships that could be construed as a potential conflict of interest.

## Publisher's Note

All claims expressed in this article are solely those of the authors and do not necessarily represent those of their affiliated organizations, or those of the publisher, the editors and the reviewers. Any product that may be evaluated in this article, or claim that may be made by its manufacturer, is not guaranteed or endorsed by the publisher.

## References

[B1] Acosta M. C. Premoli A. C. (2010). Evidence of chloroplast capture in South American Nothofagus (subgenus Nothofagus, Nothofagaceae). Mol. Phylogenet. Evol. 54, 235–242. 10.1016/j.ympev.2009.08.008 19683588

[B2] Aguilar D. L. Acosta M. C. Baranzelli M. C. Sérsic A. N. Delatorre-Herrera J. Verga A.. (2020). Ecophylogeography of the disjunct South American xerophytic tree species *Prosopis chilensis* (Fabaceae). Biol. J. Linnean Soc. 129, 793–809. 10.1093/biolinnean/blaa006

[B3] Antonelli A. Nylander J. A. A. Persson C. Sanmartin I. (2009). Tracing the impact of the Andean uplift on Neotropical plant evolution. Proc. Nat. Acad. Sci. U.S.A. 106, 9749–9754. 10.1073/pnas.0811421106 19470489 PMC2685738

[B4] Azpilicueta M. M. Marchelli P. Gallo L. A. (2009). The effects of Quaternary glaciations in Patagonia as evidenced by chloroplast DNA phylogeography of Southern beech Nothofagus obliqua. Tree Genet. Genomes 5, 561–571. 10.1007/s11295-009-0209-x

[B5] Ball P. W. Reznicek A. A. (2002). “Carex” in Flora of North America 23. eds. Editorial Committee. New York, NY: Oxford University Press.

[B6] Barreda V. Palazzesi L. (2007). Patagonian vegetation turnovers during the paleogene-early neogene: origin of arid-adapted floras. Botanical Rev. 73, 31–50. 10.1663/0006-8101(2007)73<31:PVTDTP>2.0.CO;2

[B7] Barrett P. J. (2013). Resolving views on Antarctic Neogene glacial history - the Sirius debate. Earth Environ. Sci. Trans. R. Soc. Edinb. 104, 31–53. 10.1017/S175569101300008X

[B8] Barros M. (1935). “Ciperáceas argentinas II, géneros Kyllingia, Scirpus, Carex” Anales del Museo Argentino de Ciencias Naturales. Buenos Aires.

[B9] Barros M. (1948). Una especie nueva de *Carex*. Darwiniana 8, 409–410.

[B10] Barros M. (1950). Una ciperácea y dos juncáceas nuevas. Lilloa 23, 415–420.

[B11] Barros M. (1957). Notas sobre Carex. Boletín Soc. Argent. Botánica 6, 207–211.

[B12] Barros M. (1969). Cyperaceae, in Flora Patagónica, Parte II, Typhaceae a Orchidaceae (exceptio Gramineae), ed Correa M. N. (Buenos Aires: Colección Científica del Instituto Nacional de Tecnología Agropecuaria).

[B13] Bell C. Donoghue M. J. (2005). Phylogeny and biogeography of Valerianaceae (Dipsacales) with special reference to the South American valerians. Organ. Diversity Evolut. 5, 147–159. 10.1016/j.ode.2004.10.014

[B14] Bell C. D. Kutschker A. Arroyo M. T. K. (2012). Phylogeny and diversification of Valerianaceae (Dipsacales) in the southern Andes. Mol. Phylogenet. Evol. 63, 724–737. 10.1016/j.ympev.2012.02.015 22421085

[B15] Benítez-Benítez C. Escudero M. Rodríguez-Sánchez F. Martín-Bravo S. Jiménez-Mejías P. (2018). Pliocene-Pleistocene ecological niche evolution shapes the phylogeography of a Mediterranean plant group. Mol. Ecol. 27, 1696–1713. 10.1111/mec.14567 29577497

[B16] Benítez-Benítez C. Martín-Bravo S. Bjorå C. S. Gebauer S. Hipp A. L. Hoffmann M. H.. (2021). Geographical vs. ecological diversification in Carex section Phacocystis (Cyperaceae): patterns hidden behind a twisted taxonomy. J. Syste. Evolut. 59, 642–667. 10.1111/jse.12731

[B17] Biffin E. Hill R. S. Lowe A. J. (2010). Did Kauri (*Agathis*: Araucariaceae) Really survive the Oligocene drowning of New Zealand? Syst. Biol. 59, 594–602. 10.1093/sysbio/syq030 20530131

[B18] Blomberg S. P. Rathnayake S. I. Moreau C. M. (2020). Beyond Brownian Motion and the Ornstein-Uhlenbeck process: Stochastic diffusion models for the evolution of quantitative characters. Am. Nat. 195, 145–165. 10.1086/706339 32017624

[B19] Borges P. A. V. Abreu C. Aguiar A. M. F. Carvalho P. Jardim R. Melo I.. (2008). A list of the terrestrial Fungi, Flora and Fauna of Madeira and selvagens archipelagos. Portugal: Direccão Regional do Ambiente da Madeira and Universidade dos Açores.

[B20] Breitman M. F. Avila L. J. Sites J. W. Morando M. (2012). How lizards survived blizzards: Phylogeography of the *Liolaemus lineomaculatus* group (Liolaemidae) reveals multiple breaks and refugia in southern Patagonia and their concordance with other codistributed taxa. Mol. Ecol. 21, 6068–6085. 10.1111/mec.12075 23094955

[B21] Broennimann O. Fitzpatrick M. C. Pearman P. B. Petitpierre B. Pellissier L. Yoccoz N. G.. (2012). Measuring ecological niche overlap from occurrence and spatial environmental data: Measuring niche overlap. Global Ecol. Biogeograp. 21, 481–497. 10.1111/j.1466-8238.2011.00698.x

[B22] Burnham K. P. Anderson D. R. (2002). Model Selection and Multimodel Inference: a Practical Information-Theoretical Approach. New York, NY: Springer.

[B23] Cadena C. D. Cuervo A. M. Céspedes L. N. Bravo G. A. Krabbe N. Schulenberg T. S.. (2020). Systematics, biogeography, and diversification of Scytalopus tapaculos (Rhinocryptidae), an enigmatic radiation of Neotropical montane birds. The Auk. 137:ukz077. 10.1093/auk/ukz077

[B24] Calderón L. Quintana F. Cabanne G. S. Lougheed S. C. Tubaro P. L. (2014). Phylogeography and genetic structure of two Patagonian shag species (Aves: Phalacrocoracidae). Mol. Phylogenet. Evol. 72, 42–53. 10.1016/j.ympev.2013.12.011 24418531

[B25] Cantrill D. J. Poole I. (2012). The Vegetation of antarctica Through Geological Time. Cambridge: Cambridge University Press.

[B26] Cosacov A. Johnson L. A. Paiaro V. Cocucci A. A. Córdoba F. E. Sérsic A. N. (2013). Precipitation rather than temperature influenced the phylogeography of the endemic shrub *Anarthrophyllum desideratum* in the Patagonian steppe. J. Biogeogr. 40, 168–182. 10.1111/j.1365-2699.2012.02776.x

[B27] Cosacov A. Sérsic A. N. Sosa V. Johnson L. A. Cocucci A. A. (2010). Multiple periglacial refugia in the Patagonian steppe and post-glacial colonization of the Andes: The phylogeography of *Calceolaria polyrhiza*: Phylogeography of Calceolaria polyrhiza. J. Biogeogr. 37, 1463–1477. 10.1111/j.1365-2699.2010.02307.x

[B28] Crisp M. D. Cook L. G. (2005). Do early branching lineages signify ancestral traits? Trends Ecol. Evol. 20, 122–128. 10.1016/j.tree.2004.11.010 16701355

[B29] Curtis W. M. Morris D. I. (1994). The Student's Flora of Tasmania, Part 4B. Hobart: Government Printer.

[B30] Cussac V. Ortubay S. Iglesias G. Milano D. Lattuca M. E. Barriga J. P.. (2004). The distribution of South American galaxiid fishes: The role of biological traits and post-glacial history: The distribution of South American galaxiid fishes. J. Biogeogr. 31, 103–121. 10.1046/j.0305-0270.2003.01000.x

[B31] Darwin C. D. (1859). On the Origin of Species. London: John Murray.

[B32] de la Estrella M. Buerki S. Vasconcelos T. Lucas E. J. Forest F. (2019). The role of antarctica in biogeographical reconstruction: a point of view. Int. J. Plant Sci. 180, 63–71. 10.1086/700581

[B33] Di Cola V. Broennimann O. Petitpierre B. Breiner F. T. D'Amen M. Randin C.. (2017). Ecospat: An R package to support spatial analyses and modeling of species niches and distributions. Ecography 40, 774–787. 10.1111/ecog.02671

[B34] Edgar E. (1970). Cyperaceae, in Flora of New Zealand. Vol. II. Indigenous Tracheophyta: Monocotyledones except Gramineae, eds Moore L. B. Edgar E. (Wellington: Botany Division DSIR).

[B35] Edgar R. C. (2004). MUSCLE: Multiple sequence alignment with high accuracy and high throughput. Nucleic Acids Res. 32, 1792–1797. 10.1093/nar/gkh340 15034147 PMC390337

[B36] Egorova T. V. (1999). The sedges (Carex L.) of Russia and adjacent states (within the limits of the former USSR). St. Louis: St. Petersburg State Chemical-Pharmaceutical Academy and Missouri Botanical Garden.

[B37] Encinas A. Folguera A. Bechis F. Finger K. L. Zambrano P. Pérez F.. (2018). The Late Oligocene-Early Miocene marine transgression of Patagonia, in The Evolution of the Chilean-Argentinean Andes, ed. Folguera A. Contreras Reyes E. Heredia N. Encinas A. Iannelli S. Oliveros V.. (Cham: Springer).

[B38] Ezcurra C. (2002). Phylogeny, morphology, and biogeography of chuquiraga, an andean-patagonian genus of asteraceae-barnadesioideae. Botanical Rev. 68, 153–170. 10.1663/0006-8101(2002)068<0153:PMABOC>2.0.CO;2

[B39] Fick S. E. Hijmans R. J. (2017). WorldClim 2: New 1-km spatial resolution climate surfaces for global land areas. Int. J. Climatol. 37, 4302–4315. 10.1002/joc.5086

[B40] Frajman B. Schönswetter P. Weiss-Schneeweiss H. Oxelman B. (2018). Origin and Diversification of South American Polyploid *Silene* Sect. *Physolychnis (Caryophyllaceae)* in the Andes and Patagonia. Front. Genet. 9:639. 10.3389/fgene.2018.00639 30619464 PMC6297176

[B41] Gehrke B. (2011). Synopsis of *Carex* (Cyperaceae) from sub-Saharan Africa and Madagascar. Botan. J. Linnean Soc. 166, 51–99. 10.1111/j.1095-8339.2011.01116.x

[B42] Gehrke B. Martín-Bravo S. Muasya M. Luceño M. (2010). Monophyly, phylogenetic position and the role of hybridization in *Schoenoxiphium* Nees (Cariceae, Cyperaceae). Mol. Phylogenet. Evol. 56, 380–392. 10.1016/j.ympev.2010.03.036 20363346

[B43] Givnish T. J. Millam K. C. Evans T. M. Hall J. C. Chris Pires J. Berry P. E.. (2004). Ancient vicariance or Recent Long-Distance Dispersal? Inferences about phylogeny and South American–African disjunctions in Rapateaceae and Bromeliaceae based on ndh F sequence data. Int. J. Plant Sci. 165, S35–S54. 10.1086/421067

[B44] Glenny D. (2004). A revision of the genus *Gentianella* in New Zealand. NZ. J. Bot. 42, 361–530. 10.1080/0028825X.2004.9512910

[B45] Govaerts R. Jiménez-Mejías P. Koopman J. Simpson D. Goetghebeur P. Wilson K.. (2020). World Checklist of Cyperaceae. Available online at: http://wcsp.science.kew.org/ (accessed July 16, 2020).

[B46] Hamlin B. G. (1962). Studies in New Zealand Carices - VI. Subgenus Primocarex Kükenthal. Transact. R. Soc. New Zeal. Botany 1, 269–277.

[B47] Harmon L. J. Weir J. T. Brock C. D. Glor R. E. Challenger W. (2008). GEIGER: Investigating evolutionary radiations. Bioinformatics 24, 129–131. 10.1093/bioinformatics/btm538 18006550

[B48] He X. Sun Y. (2017). Contrasting patterns of postglacial range shifts between the northern and southern hemisphere in *Herbertus* (Herbertaceae, Marchantiophyta). System. Biodivers. 15, 541–551. 10.1080/14772000.2017.1291542

[B49] Heenan P. B. McGlone M. S. (2013). Evolution of New Zealand alpine and open-habitat plant species during the late Cenozoic. New Zeal. J. Ecol. 37, 105–113. Available online at: https://newzealandecology.org/nzje/

[B50] Heibl C. Renner S. S. (2012). Distribution models and a dated phylogeny for Chilean *Oxalis* species reveal occupation of new habitats by different lineages, not rapid adaptive radiation. Syst. Biol. 61, 823–834. 10.1093/sysbio/sys034 22357726

[B51] Hewitt G. M. (2000). The genetic legacy of the Quaternary ice ages. Nature 405, 907–913. 10.1038/35016000 10879524

[B52] Hewitt G. M. (2004). Genetic consequences of climatic oscillations in the Quaternary. Philosoph. Transact. R. Soc. Lond. Series B Biol. Sci. 359, 183–195. 10.1098/rstb.2003.1388 15101575 PMC1693318

[B53] Holderegger R. Thiel-Egenter C. (2009). A discussion of different types of glacial refugia used in mountain biogeography and phylogeography. J. Biogeogr. 36, 476–480. 10.1111/j.1365-2699.2008.02027.x

[B54] Hooker J. D. (1853). Introductory Essay to the Flora of New Zealand. London: Reeve.

[B55] Hughes C. Eastwood R. (2006). Island radiation on a continental scale: Exceptional rates of plant diversification after uplift of the Andes. Proc. Nat. Acad. Sci. U.S.A. 103, 10334–10339. 10.1073/pnas.0601928103 16801546 PMC1502458

[B56] Jakob S. S. Martinez-Meyer E. Blattner F. R. (2009). Phylogeographic analyses and paleodistribution modeling indicate Pleistocene *in situ* survival of Hordeum species (Poaceae) in southern Patagonia without genetic or spatial restriction. Mol. Biol. Evol. 26, 907–923. 10.1093/molbev/msp012 19168565

[B57] Jiménez-Mejías P. Alegría-Olivera J. J. Beltrán H. W. Cano A. Granda-Paucar A. Maldonado Fonkén M. S.. (2020). Chorological and nomenclatural notes on Peruvian *Carex* (Cyperaceae). Caldasia 42, 63–69. 10.15446/caldasia.v42n1.76771

[B58] Jiménez-Mejías P. Benítez-Benítez C. Beltrán H. Cano Echevarría A. Donadío S. Escudero M.. (2018). Carex (Cyperaceae) in South America: Diversity, Phylogenetics and Biogeography of a Boreotemperate Element in the Neotropics. [Poster presented at the 6^th^ International Conference on Comparative Biology of Monocotyledons]. [Natal, Rio Grande do Norte, Brazil].

[B59] Jiménez-Mejías P. Hahn M. Lueders K. Starr J. R. Brown B. H. Chouinard B. N.. (2016a). Megaphylogenetic specimen-level approaches to the *Carex* (Cyperaceae) phylogeny using ITS, ETS, and mat*K*: Implications for classification. Syst. Bot. 41, 500–518. 10.1600/036364416X692497

[B60] Jiménez-Mejías P. Luceño M. (2011). Cyperaceae, in Euro+Med Plantbase - The Information Resource for Euro-Mediterranean Plant Diversity. Available online at: http://ww2.bgbm.org/EuroPlusMed/query.asp

[B61] Jiménez-Mejías P. Martinetto E. Momohara A. Popova S. Smith S. Y. Roalson E. H. (2016b). A commented synopsis of the Pre-Pleistocene fossil record of *Carex* (Cyperaceae). Botanical Rev. 82, 258–345. 10.1007/s12229-016-9169-7

[B62] Jiménez-Mejías P. Roalson E. H. (2016). Two new species of *Carex* (Cyperaceae) from northern South America. Phytotaxa 266:21. 10.11646/phytotaxa.266.1.3

[B63] Kim I. Phillips C. J. Monjeau J. A. Birney E. C. Noack K. Pumo D. E.. (1998). Habitat islands, genetic diversity, and gene flow in a Patagonian rodent. Mol. Ecol. 7, 667–678. 10.1046/j.1365-294x.1998.00369.x 9640647

[B64] Knapp M. Stöckler K. Havell D. Delsuc F. Sebastiani F. Lockhart P. J. (2005). Relaxed molecular clock provides evidence for long-distance dispersal of nothofagus (Southern Beech). PLoS Biol. 3:e14. 10.1371/journal.pbio.0030014 15660155 PMC539330

[B65] Koenig K. (2016). Biodiversity hotspots map (English labels). Zenodo. 10.5281/zenodo.4311850

[B66] Kozak K. H. Wiens J. J. (2006). Does niche conservatism promote speciation? A case study in North American salamanders. Evolution 60, 2604–2621. 10.1111/j.0014-3820.2006.tb01893.x 17263120

[B67] Kranitz M. L. Biffin E. Clark A. Hollingsworth M. L. Ruhsam M. Gardner M. F.. (2014). Evolutionary diversification of new caledonian araucaria. PLoS ONE 9:e110308. 10.1371/journal.pone.0110308 25340350 PMC4207703

[B68] Kremer A. (2016). Microevolution of European temperate oaks in response to environmental changes. C. R. Biol. 339, 263–267. 10.1016/j.crvi.2016.04.014 27263361

[B69] Landis M. J. Matzke N. J. Moore B. R. Huelsenbeck J. P. (2013). Bayesian analysis of biogeography when the number of areas is large. Syst. Biol. 62, 789–804. 10.1093/sysbio/syt040 23736102 PMC4064008

[B70] Lewis A. R. Marchant D. R. Ashworth A. C. Hedenas L. Hemming S. R. Johnson J. V.. (2008). Mid-Miocene cooling and the extinction of tundra in continental Antarctica. Proc. Nat. Acad. Sci. U.S.A. 105, 10676–10680. 10.1073/pnas.0802501105 18678903 PMC2495011

[B71] López A. Bonasora M. G. (2017). Phylogeography, genetic diversity and population structure in a Patagonian endemic plant. AoB PLANTS 9:plx017. 10.1093/aobpla/plx017 28567240 PMC5442505

[B72] Losos J. B. (2008). Phylogenetic niche conservatism, phylogenetic signal and the relationship between phylogenetic relatedness and ecological similarity among species. Ecol. Lett. 11, 995–1003. 10.1111/j.1461-0248.2008.01229.x 18673385

[B73] Luceño M. (2008). Carex, in Flora Iberica, eds Castroviejo S. Luceño M. Galán de Mera A. Jiménez-Mejías P. Cabezas F. J. Medina L. (Madrid: Real Jardín Botánico).

[B74] Luceño M. Villaverde T. Márquez-Corro J. I. Sánchez-Villegas R. Maguilla E. Escudero M.. (2021). An integrative monograph of *Carex* section *Schoenoxiphium* (Cyperaceae). PeerJ 9:e11336. 10.7717/peerj.11336 34046256 PMC8136282

[B75] Luebert F. Hilger H. H. Weigend M. (2011). Diversification in the andes: age and origins of South American heliotropium lineages (Heliotropiaceae, Boraginales). Mol. Phylogenet. Evol. 61, 90–102. 10.1016/j.ympev.2011.06.001 21689768

[B76] Luebert F. Lörch M. Acuña R. Mello-Silva R. Weigend M. Mutke J. (2020). Clade-specific biogeographic history and climatic niche shifts of the southern Andean-southern Brazilian disjunction in plants, in Neotropical Diversification: Patterns and Processes, eds Rull V. Carnaval A. C. (Cham: Springer International Publishing). 10.1007/978-3-030-31167-4_24

[B77] Mairal M. Sanmartín I. Pellissier L. (2017). Lineage-specific climatic niche drives the tempo of vicariance in the Rand Flora. J. Biogeogr. 44, 911–923. 10.1111/jbi.12930

[B78] Marcheli P. Gallo L. (2006). Multiple ice-age refugia in a southern beech of South America as evidenced by chloroplast DNA markers. Conserv. Genet. 7, 591–603. 10.1007/s10592-005-9069-6

[B79] Markgraf V. (1983). Late and postglacial vegetational and paleoclimatic changes in subantarctic, temperate, and arid environments in Argentina. Palynology 7, 43–70. 10.1080/01916122.1983.9989252

[B80] Markgraf V. McGlone M. Hope G. (1995). Neogene paleoenvironmental and paleoclimatic change in southern temperate ecosystems—A southern perspective. Trends Ecol. Evol. 10, 143–147. 10.1016/S0169-5347(00)89023-0 21236983

[B81] Márquez-Corro J. I. Jiménez-Mejías P. Helme N. A. Luceño M. Martín-Bravo S. (2020). The systematic position of the enigmatic rare South African endemic *Carex acocksii*: Its relevance on the biogeography and evolution of *Carex* sect. Schoenoxiphium (Cyperaceae). South Afr. J. Botany 131, 475–483. 10.1016/j.sajb.2020.03.027

[B82] Martín-Bravo S. Jiménez-Mejías P. Villaverde T. Escudero M. Hahn M. Spalink D.. (2019). A tale of worldwide success: Behind the scenes of *Carex* (Cyperaceae) biogeography and diversification. J. Syst. Evol. 57, 695–718. 10.1111/jse.12549

[B83] Martínez O. A. Kutschker A. (2011). The ‘Rodados Patagónicos’ (Patagonian shingle formation) of eastern Patagonia: Environmental conditions of gravel sedimentation: Patagonian gravels in eastern Patagonia. Biol. J. Linnean Soc. 103, 336–345. 10.1111/j.1095-8312.2011.01651.x

[B84] Mathiasen P. Premoli A. C. (2010). Out in the cold: Genetic variation of *Nothofagus pumilio* (Nothofagaceae) provides evidence for latitudinally distinct evolutionary histories in austral South America: Genetic structure of N. pumilio. Mol. Ecol. 19, 371–385. 10.1111/j.1365-294X.2009.04456.x 20002584

[B85] Matzke N. J. (2014a). BioGeoBEARS: BioGeography With Bayesian (and Likelihood) Evolutionary Analysis with R Scripts, v. 1.1.1. Available online at: https://rdrr.io/cran/BioGeoBEARS (accessed November 15, 2020).

[B86] Matzke N. J. (2014b). Model selection in historical biogeography reveals that founder-event speciation is a crucial process in Island clades. Syst. Biol. 63, 951–970. 10.1093/sysbio/syu056 25123369

[B87] McGlone M. S. Duncan R. P. Heenan P. B. (2001). Endemism, species selection and the origin and distribution of the vascular plant flora of New Zealand: Origin and distribution of the New Zealand flora. J. Biogeograp. 28, 199–216. 10.1046/j.1365-2699.2001.00525.x

[B88] McLoughlin S. (2001). The breakup history of Gondwana and its impact on pre-Cenozoic floristic provincialism. Aust. J. Bot. 49:271. 10.1071/BT00023

[B89] Meudt H. M. Simpson B. B. (2006). The biogeography of the austral, subalpine genus *Ourisia* (Plantaginaceae) based on molecular phylogenetic evidence: South American origin and dispersal to New Zealand and Tasmania. Biol. J. Linnean Soc. 87, 479–513. 10.1111/j.1095-8312.2006.00584.x

[B90] Míguez M. Gehrke B. Maguilla E. Jiménez-Mejías P. Martín-Bravo S. (2017). *Carex* sect. *Rhynchocystis* (Cyperaceae): A miocene subtropical relict in the Western Palaearctic showing a dispersal-derived Rand Flora pattern. J. Biogeogr. 44, 2211–2224. 10.1111/jbi.13027

[B91] Miller M. A. Pfeiffer W. Schwartz T. (2010). Creating the CIPRES science gateway for inference of large phylogenetic trees, in Proceedings of the Gateway Computing Environments Workshop, 14, 1–8. 10.1109/GCE.2010.5676129

[B92] Moore D. M. (1968). The vascular flora of the Falkland Islands. Br. Antarct. Sur. Sci. Rep. 60, 1–202.

[B93] Moore D. M. (1983). Flora of Tierra del Fuego. Oswestry; St. Louis, MO: Anthony Nelson Ltd. and Missouri Botanical Garden.

[B94] Moore L. B. Edgar E. (1970). Flora of New Zealand Volume II. Wellington: Indigenous Tracheophyta, Monocotyledons except Gramineae. Government Printer.

[B95] Mora A. Villagómez D. Parra M. Caballero V. M. Spikings R. Horton B. K.. (2020). Late Cretaceous to Cenozoic uplifts of the Northern Andes: Paleogeographic implications, in The Geology of Colombia, eds Gómez J. Mateus-Zabala D. (Bogotá: Servicio Geológico Colombiano, Publicaciones Geológicas Especiales). 10.32685/pub.esp.37.2019.04

[B96] Morando M. Avila L. J. Baker J. Sites J. W. (2004). Phylogeny and phylogeography of the *Liolaemus darwinii* complex (Squamata: Liolaemidae): evidence for introgression and incomplete lineage sorting. Evolution 58, 842–859. 10.1111/j.0014-3820.2004.tb00416.x 15154559

[B97] Morando M. Avila L. J. Turner C. R. Sites J. W. (2007). Molecular evidence for a species complex in the patagonian lizard *Liolaemus bibronii* and phylogeography of the closely related *Liolaemus gracilis* (Squamata: Liolaemini). Mol. Phylogenet. Evol. 43, 952–973. 10.1016/j.ympev.2006.09.012 17116410

[B98] Morello S. Sede S. M. (2016). Genetic admixture and lineage separation in a southern Andean plant. AoB Plants 8:plw034. 10.1093/aobpla/plw034 27179539 PMC4940511

[B99] Muellner A. N. Tremetsberger K. Stuessy T. Baeza C. M. (2005). Pleistocene refugia and recolonization routes in the southern Andes: Insights from Hypochaeris palustris (Asteraceae, Lactuceae): *Hypochaeris palustris* in the Southern Andes. Mol. Ecol. 14, 203–212. 10.1111/j.1365-294X.2004.02386.x 15643964

[B100] Nicola M. V. Johnson L. A. Pozner R. (2019). Unraveling patterns and processes of diversification in the South Andean-Patagonian Nassauvia subgenus Strongyloma (Asteraceae, Nassauvieae). Mol. Phylogenet. Evol. 136, 164–182. 10.1016/j.ympev.2019.03.004 30858079

[B101] Nicola M. V. Sede S. M. Pozner R. Johnson L. A. (2014). Phylogeography and palaeodistribution modelling of *Nassauvia* subgenus *Strongyloma* (Asteraceae): Exploring phylogeographical scenarios in the Patagonian steppe. Ecol. Evol. 4, 4270–4286. 10.1002/ece3.1268 25540689 PMC4267866

[B102] Noben S. Kessler M. Quandt D. Weigand A. Wicke S. Krug M.. (2017). Biogeography of the Gondwanan tree fern family Dicksoniaceae-A tale of vicariance, dispersal and extinction. J. Biogeogr. 44, 2648–2659. 10.1111/jbi.13056

[B103] Nürk N. M. Scheriau C. Madriñán S. (2013). Explosive radiation in high Andean *Hypericum*-rates of diversification among New World lineages. Front. Genet. 4:175. 10.3389/fgene.2013.00175 24062764 PMC3769627

[B104] Otero A. Jiménez-Mejías P. Valcárcel V. Vargas P. (2019). Worldwide long-distance dispersal favored by epizoochorous traits in the biogeographic history of Omphalodeae (Boraginaceae). J. Syst. Evol. 57, 579–593. 10.1111/jse.12504

[B105] Pastorino M. J. Gallo L. A. Hattemer H. H. (2004). Genetic variation in natural populations of Austrocedrus chilensis, a cypress of the Andean-Patagonian Forest. Biochem. Syst. Ecol. 32, 993–1008. 10.1016/j.bse.2004.03.002

[B106] Pastorino M. J. Marchelli P. Milleron M. Soliani C. Gallo L. A. (2009). The effect of different glaciation patterns over the current genetic structure of the southern beech *Nothofagus antarctica*. Genetica 136, 79–88. 10.1007/s10709-008-9314-2 18758967

[B107] Pearman P. B. Guisan A. Broennimann O. Randin C. F. (2008). Niche dynamics in space and time. Trends Ecol. Evol. 23, 149–158. 10.1016/j.tree.2007.11.005 18289716

[B108] Pender J. E. Hipp A. L. Hahn M. Starr J. R. (2021). Trait evolution rates shape continental patterns of species richness in North America's most diverse angiosperm genus (*Carex*, Cyperaceae). J. Syst. Evol. 59, 763–775. 10.1111/jse.12739

[B109] Ponce J. F. Rabassa J. Coronato A. Borromei A. M. (2011). Palaeogeographical evolution of the Atlantic coast of Pampa and Patagonia from the last glacial maximum to the Middle Holocene: Palaeogeography of Patagonia since LGM. Biol. J. Linnean Soc. 103, 363–379. 10.1111/j.1095-8312.2011.01653.x

[B110] Premoli A. C. Kitzberger T. Veblen T. T. (2000). Isozyme variation and recent biogeographical history of the long-lived conifer *Fitzroya cupressoides*. J. Biogeogr. 27, 251–260. 10.1046/j.1365-2699.2000.00402.x

[B111] Premoli A. C. Mathiasen P. Kitzberger T. (2010). Southernmost *Nothofagus* trees enduring ice ages: Genetic evidence and ecological niche retrodiction reveal high latitude (54°S) glacial refugia. Palaeogeogr. Palaeoclimatol. Palaeoecol. 298, 247–256. 10.1016/j.palaeo.2010.09.030

[B112] Quiroga M. P. Premoli A. C. (2010). Genetic structure of *Podocarpus nubigena* (Podocarpaceae) provides evidence of Quaternary and ancient historical events. Palaeogeogr. Palaeoclimatol. Palaeoecol. 285, 186–193. 10.1016/j.palaeo.2009.11.010

[B113] R Development Core Team (2019). R: A LANGUAGE and Environment for Statistical Computing. Austria: R Foundation for Statistical Computing. Available online at: https://www.r-project.org/ (accessed November 20, 2020).

[B114] Rabassa J. (2008). Late cenozoic glaciations in Patagonia and Tierra del Fuego, in Developments in Quaternary Sciences, Vol. 11 (Amsterdam: Elsevier). 10.1016/S1571-0866(07)10008-7

[B115] Rabassa J. Coronato A. Martínez O. (2011). Late Cenozoic glaciations in Patagonia and Tierra del Fuego: An updated review: Late Cenozoic Patagonian glaciations. Biol. J. Linnean Soc. 103, 316–335. 10.1111/j.1095-8312.2011.01681.x

[B116] Rabassa J. Coronato A. M. Salemme M. (2005). Chronology of the Late Cenozoic Patagonian glaciations and their correlation with biostratigraphic units of the Pampean region (Argentina). J. South Am. Earth Sci. 20, 81–103. 10.1016/j.jsames.2005.07.004

[B117] Rabosky D. L. Grundler M. Anderson C. Title P. Shi J. J. Brown J. W.. (2014). BAMMtools: An R package for the analysis of evolutionary dynamics on phylogenetic trees. Methods Ecol. Evolution 5, 701–707. 10.1111/2041-210X.12199

[B118] Ramos V. A. Ghiglione M. C. (2008). Tectonic evolution of the patagonian Andes, in Developments in Quaternary Sciences, Vol. 11 (Amsterdam: Elsevier). 10.1016/S1571-0866(07)10004-X

[B119] Ree R. H. Sanmartín I. (2018). Conceptual and statistical problems with the DEC+J model of founder-event speciation and its comparison with DEC via model selection. J. Biogeograp. 45, 741–749. 10.1111/jbi.13173

[B120] Ree R. H. Smith S. A. (2008). Maximum likelihood inference of geographic range evolution by dispersal, local extinction, and cladogenesis. Syst. Biol. 57, 4–14. 10.1080/10635150701883881 18253896

[B121] Revell L. J. (2012). Phytools: An R package for phylogenetic comparative biology (and other things): phytools: R package. Methods Ecol. Evolut. 3, 217–223. 10.1111/j.2041-210X.2011.00169.x

[B122] Roalson E. H. Jiménez-Mejías P. Hipp A. L. Benítez-Benítez C. Bruederle L. P. Chung K.-S.. (2021). A framework infrageneric classification of *Carex* (Cyperaceae) and its organizing principles. J. Syst. Evol. 59, 726–762. 10.1111/jse.12722

[B123] Roberts N. J. Barendregt R. W. Clague J. J. (2017). Multiple tropical Andean glaciations during a period of late Pliocene warmth. Sci. Rep. 7:41878. 10.1038/srep41878 28169346 PMC5294413

[B124] Ronquist F. (1997). Dispersal-Vicariance analysis: a new approach to the quantification of historical biogeography. Syst. Biol. 46:9.

[B125] Ronquist F. Teslenko M. van der Mark P. Ayres D. L. Darling A. Höhna S.. (2012). MrBayes 3.2: efficient bayesian phylogenetic inference and model choice across a large model space. Syst. Biol. 61, 539–542. 10.1093/sysbio/sys029 22357727 PMC3329765

[B126] Rull V. (2008). Speciation timing and neotropical biodiversity: The Tertiary-Quaternary debate in the light of molecular phylogenetic evidence: Speciation timing and neotropical biodiversity. Mol. Ecol. 17, 2722–2729. 10.1111/j.1365-294X.2008.03789.x 18494610

[B127] Ruzzante D. E. Walde S. J. Gosse J. C. Cussac V. E. Habit E. Zemlak T. S.. (2008). Climate control on ancestral population dynamics: Insight from Patagonian fish phylogeography: Historical population dynamics of Patagonian fish. Mol. Ecol. 17, 2234–2244. 10.1111/j.1365-294X.2008.03738.x 18363661

[B128] Sanmartín I. Ronquist F. (2004). Southern hemisphere biogeography inferred by event-based models: plant versus animal patterns. Syst. Biol. 53, 216–243. 10.1080/10635150490423430 15205050

[B129] Schoener T. W. (1968). The anolis lizards of bimini: resource partitioning in a complex fauna. Ecology 49, 704–726. 10.2307/1935534

[B130] Sede S. M. Nicola M. V. Pozner R. Johnson L. A. (2012). Phylogeography and palaeodistribution modelling in the Patagonian steppe: The case of Mulinum spinosum (Apiaceae): Phylogeography and palaeodistribution modelling of *Mulinum spinosum*. J. Biogeogr. 39, 1041–1057. 10.1111/j.1365-2699.2011.02662.x

[B131] Segovia R. A. Pérez M. F. Hinojosa L. F. (2012). Genetic evidence for glacial refugia of the temperate tree *Eucryphia cordifolia* (Cunoniaceae) in southern South America. Am. J. Bot. 99, 121–129. 10.3732/ajb.1100013 22210838

[B132] Sérsic A. N. Cosacov A. Cocucci A. A. Johnson L. A. Pozner R. Avila L. J.. (2011). Emerging phylogeographical patterns of plants and terrestrial vertebrates from Patagonia: Phylogeographical patterns from Patagonia. Biol. J. Linnean Soc. 103, 475–494. 10.1111/j.1095-8312.2011.01656.x

[B133] Shaw J. Lickey E. B. Beck J. T. Farmer S. B. Liu W. Miller J.. (2005). The tortoise and the hare II: Relative utility of 21 noncoding chloroplast DNA sequences for phylogenetic analysis. Am. J. Bot. 92, 142–166. 10.3732/ajb.92.1.142 21652394

[B134] Silva L. Moura M. Schaefer H. Rumsey F. Dias E. F. (2010). List of vascular plants (Tracheobionta), in A List of the Terrestrial and Marine Biota From the Azores, eds Borges P. A. V. Costa A. Cunha R. Gabriel R. Gonçalves V. Martins A. F.. (Cascáis: Principia) 117–146.

[B135] Silveira G. H. Longui-Wagner H. M. (2012). O gênero *Carex* L. (Cyperaceae) no Rio Grande do Sul, Brasil. Brazil. J. Biosci. 10, 373–417.

[B136] Simmons M. P. Ochoterena H. (2000). Gaps as characters in sequence-based phylogenetic analyses. Syst. Biol. 49, 369–381. 10.1093/sysbio/49.2.369 12118412

[B137] Simões M. Breitkreuz L. Alvarado M. Baca S. Cooper J. C. Heins L.. (2016). The evolving theory of evolutionary radiations. Trends Ecol. Evol. 31, 27–34. 10.1016/j.tree.2015.10.007 26632984

[B138] Simpson B. B. Tate J. A. Weeks A. (2005). The biogeography of Hoffmannseggia (Leguminosae, Caesalpinioideae, Caesalpinieae): A tale of many travels: The biogeography of Hoffmannseggia, Leguminosae. J. Biogeogr. 32, 15–27. 10.1111/j.1365-2699.2004.01161.x

[B139] Simpson M. G. Johnson L. A. Villaverde T. Guilliams C. M. (2017). American amphitropical disjuncts: Perspectives from vascular plant analyses and prospects for future research. Am. J. Bot. 104, 1600–1650. 10.3732/ajb.1700308

[B140] Slingsby J. A. Verboom G. A. (2006). Phylogenetic relatedness limits co-occurrence at fine spatial scales: evidence from the Schoenoid sedges (Cyperaceae: Schoeneae) of the Cape Floristic Region, South Africa. Am. Nat. 168, 14–27. 10.1086/505158 16874612

[B141] Soliani C. Tsuda Y. Bagnoli F. Gallo L. A. Vendramin G. G. Marcella P. (2015). Halfway encounters: Meeting points of colonization routes among the southern beeches Nothofagus pumilio and N. antarctica. Mol. Phylogenet. Evolut. 85, 197–207. 10.1016/j.ympev.2015.01.006 25639456

[B142] Souto C. P. Kitzberger T. Arbetman M. P. Premoli A. C. (2015). How do cold-sensitive species endure ice ages? Phylogeographic and paleodistribution models of postglacial range expansion of the mesothermic drought-tolerant conifer Austrocedrus chilensis. New Phytol. 208, 960–972. 10.1111/nph.13508 26079667

[B143] Souto C. P. Premoli A. C. (2007). Genetic variation in the widespread *Embothrium coccineum* (Proteaceae) endemic to Patagonia: Effects of phylogeny and historical events. Aust. J. Bot. 55, 809–817. 10.1071/BT06183

[B144] Spalik K. Piwczyński M. Danderson C. A. Kurzyna-Młynik R. Bone T. S. Downie S. R. (2010). Amphitropic amphiantarctic disjunctions in Apiaceae subfamily Apioideae. J. Biogeogr. 37, 1977–1994. 10.1111/j.1365-2699.2010.02334.x

[B145] Spalink D. Drew B. T. Pace M. C. Zaborsky J. G. Starr J. R. Cameron K. M.. (2016). Biogeography of the cosmopolitan sedges (Cyperaceae) and the area-richness correlation in plants. J. Biogeogr. 43, 1893–1904. 10.1111/jbi.12802

[B146] Specht C. (2006). Gondwanan Vicariance or Dispersal in the Tropics? The biogeographic history of the tropical monocot family costaceae (Zingiberales). Aliso 22, 633–644. 10.5642/aliso.20062201.50

[B147] Stamatakis A. (2014). RAxML v. 8: A tool for phylogenetic analysis and post-analysis of large phylogenies. Bioinformatics 30, 1312–1313. 10.1093/bioinformatics/btu033 24451623 PMC3998144

[B148] Suchard M. A. Lemey P. Baele G. Ayres D. L. Drummond A. J. Rambaut A. (2018). Bayesian phylogenetic and phylodynamic data integration using BEAST 1.10. Virus Evolut. 4:16. 10.1093/ve/vey016 29942656 PMC6007674

[B149] Title P. O. Bemmels J. B. (2018). ENVIREM: An expanded set of bioclimatic and topographic variables increases flexibility and improves performance of ecological niche modeling. Ecography 41, 291–307. 10.1111/ecog.02880

[B150] Tremetsberger K. Urtubey E. Terrab A. Baeza C. M. Ortiz M. Á. Talavera M.. (2009). Pleistocene refugia and polytopic replacement of diploids by tetraploids in the Patagonian and Subantarctic plant *Hypochaeris incana* (Asteraceae, Cichorieae). Mol. Ecol. 18, 3668–3682. 10.1111/j.1365-294X.2009.04298.x 19674310

[B151] Vargas P. Zardoya R. (2014). The Tree of Life: Evolution and Classification of Living Organisms. Massachussets: Sinauer.

[B152] Vera-Escalona I. D'Elía G. Gouin N. Fontanella F. M. Muñoz-Mendoza C. Sites J. W.. (2012). Lizards on ice: evidence for multiple refugia in *Liolaemus pictus* (Liolaemidae) during the last glacial maximum in the southern andean beech forests. PLoS ONE 7:e48358. 10.1371/journal.pone.0048358 23209552 PMC3507886

[B153] Verdcourt B. (2010). Carex, in Flora of Tropical East Africa, eds Beentje H. J. Ghazanfar S. A. (London: Richmond Kew Publishing).

[B154] Vidal-Russell R. Souto C. P. Premoli A. C. (2011). Multiple Pleistocene refugia in the widespread Patagonian tree *Embothrium coccineum* (Proteaceae). Aust. J. Bot. 59, 299–314. 10.1071/BT10303

[B155] Villaverde T. Escudero M. Martín-Bravo S. Jiménez-Mejías P. Sanmartín I. Vargas P.. (2017a). Bipolar distributions in vascular plants: A review. Am. J. Bot. 104, 1680–1694. 10.3732/ajb.1700159 29167157

[B156] Villaverde T. Jiménez-Mejías P. Luceño M. Waterway M. J. Kim S. Lee B.. (2020). A new classification of *Carex* (Cyperaceae) subgenera supported by a HybSeq backbone phylogenetic tree. Botan. J. Linnean Soc. 194, 141–163. 10.1093/botlinnean/boaa042

[B157] Villaverde T. Maguilla E. Escudero M. Márquez-Corro J. I. Jiménez-Mejías P. Gehrke B.. (2017b). New insights into the systematics of the *Schoenoxiphium* Clade (*Carex*, Cyperaceae). Int. J. Plant Sci. 178, 320–329. 10.1086/691144

[B158] Von Hagen K. B. Kadereit J. W. (2001). The phylogeny of *Gentianella* (Gentianaceae) and its colonization of the southern hemisphere as revealed by nuclear and chloroplast DNA sequence variation. Organ. Diversity Evolut. 1, 61–79. 10.1078/1439-6092-00005

[B159] Wagstaff S. J. Bayly M. Garnock-Jones P. J. Albach D. (2002). Classification, origin, and diversification of the New Zealand Hebes (Scrophulariaceae). Ann. Missouri Botan. Garden 89, 38–63. 10.2307/3298656

[B160] Warren D. L. Glor R. E. Turelli M. (2008). Environmental niche equivalency versus conservatism: quantitative approaches to niche evolution. Evolution 62, 2868–2883. 10.1111/j.1558-5646.2008.00482.x 18752605

[B161] Wheeler G. A. (1988). The distribution of Carex acaulis Urv., *C. barrosii Nelmes*, and *C. macrosolen* Steudel (Cyperaceae) in Austral South America. Taxon 37, 127–131. 10.2307/1220939

[B162] Wheeler G. A. (1989). The taxonomy of *Carex* sect. Aciculares (Cyperaceae) in South America. System. Botany 14, 168–188. 10.2307/2418904

[B163] Wheeler G. A. (1998). Notes on *Carex azuayae* and *C. enneastachya* (Cyperaceae) from South America. Rhodora 100, 293–297.

[B164] Wheeler G. A. Beck S. G. (2011). A new combination in *Carex* (Cyperaceae) and the first report of five other Cariceae from Bolivia. Rev. Soc. Bolivi. Botán. 5, 47–52.

[B165] Wheeler G. A. Guaglianone E. R. (2003). Notes of South American *Carex* (Cyperaceae): *C. camptoglochin* and *C. microglochin*. Darwiniana 41, 193–206.

[B166] Wheeler G. A. Muñoz-Schick M. (1990). *Carex andina* Philippi (Cyperaceae): its taxonomy, distribution and lectotypification. Gayana Botan. 47, 71–76.

[B167] Wickham H. (2006). ggplot: An Implementation of the Grammar of Graphics in R Package version 2.0.0.

[B168] Wiens J. J. Ackerly D. D. Allen A. P. Anacker B. L. Buckley L. B. Cornell H. V.. (2010). Niche conservatism as an emerging principle in ecology and conservation biology. Ecol. Lett. 13, 1310–1324. 10.1111/j.1461-0248.2010.01515.x 20649638

[B169] Wiens J. J. Graham C. H. (2005). Niche conservatism: integrating evolution, ecology, and conservation biology. Annu. Rev. Ecol. Evol. Syst. 36, 519–539. 10.1146/annurev.ecolsys.36.102803.095431

[B170] Xu J. Pérez-Losada M. Jara C. G. Crandall K. A. (2009). Pleistocene glaciation leaves deep signature on the freshwater crab *Aegla alacalufi* in Chilean Patagonia. Mol. Ecol. 18, 904–918. 10.1111/j.1365-294X.2008.04070.x 19207249

[B171] Zemlak T. S. Habit E. M. Walde S. J. Battini M. A. Adams E. D. M. Ruzzante D. E. (2008). Across the southern Andes on fin: Glacial refugia, drainage reversals and a secondary contact zone revealed by the phylogeographical signal of *Galaxias platei* in Patagonia. Mol. Ecol. 17, 5049–5061. 10.1111/j.1365-294X.2008.03987.x 19017262

