# Supplementary material for: An Evolutionary Study of Carex Subg. Psyllophorae (Cyperaceae) Sheds Light on a Strikingly Disjunct Distribution in the Southern Hemisphere, With Emphasis on Its Patagonian Diversification
Source: Front Plant Sci. 2021 Nov 8;12:735302. doi: 10.3389/fpls.2021.735302 (PMC8606891; doi:10.3389/fpls.2021.735302)
Supplement: Supplementary file 1 [file Data_Sheet_1.zip › Supplementary Data 1 and 2.PDF]

SUPPLEMENTARY DATA 1

Additional methodological details

Phylogenetic reconstruction, estimation of divergence times and diversification rate analyses

Maximum Likelihood phylogenetic analysis was run using GTRCAT model and automatic bootstrapping (100 replicates). For BI, four simultaneous Markov Chain Monte Carlo (MCMC) chains were run for 10 million generations, sampling trees every 1000 generations. The best models of nucleotide evolution were selected under Akaike Information Criterion (AIC) as calculated in jModelTest v.2.1 (Darriba et al., 2012). For indels we implemented the model F81 as suggested in MrBayes's manual.

Dating analyses were conducted for both the complete tree of subg. *Psyllophorae.* Prior node age of the fossils were implemented as a lognormal distribution except for the root node calibrated with *C. colwellensis* in which we used a normal distribution to avoid a hard minimum bound at the tree root and accommodate bidirectional dating uncertainty of C. *colwellensis* fossil (*incertae sedis*; see Jiménez-Mejías et al., 2016b), following Ho and Phillips (2009). Similarly, normal distribution was chosen for the secondary calibration (Table 2). Separated models of nucleotide substitution were considered for each of the four partitions. Two uncorrelated log-normal relaxed clocks were set separately for plastid (*matK*, *rps16*; uniform distribution = 1 x 10^{-4} – 1 x 10^{-2}) and the nuclear region (ITS, ETS; uniform distribution = 5 x 10^{-4} – 5 x 10^{-2}) based on the boundaries proposed by Blanco-Pastor et al. (2012). Four independent MCMC runs with 50 million generations each were performed, with the parameters sampled every 10,000 generations. A Birth-Death tree model was set for all of the four partitions. We visualized the results in Tracer v. 1.7 (Rambaut et al., 2018), as well as run convergence and effective sample size (ESS; values considered reliable when greater than 200) were examined. Trees and parameters from the four independent runs were combined performing 25% burn-in in LogCombiner v.1.10.4. A summary of the trees (maximum clade credibility, MCC tree) was calculated with TreeAnnotator v.1.10.4 using mean heights for node ages. Finally, the resulting MCC tree were visualized in FigTree v.1.4.2 (Rambaut, 2014). A singleton chronogram was obtained by pruning the complete tree to one tip per taxa, and used for downstream diversification and biogeographic analyses. This singleton tree was further pruned to sect. *Junciformes,* in order to conduct detailed analyses for this group, including diversification, biogeographic and ancestral state reconstruction of ecological preferences.

Regarding diversification rate analyses, the R package BAMMtools (Rabosky et al., 2014) was implemented to assess the most optimal priors for configuration file, the convergence of MCMC as well as observed exchange rates. The results were visualized using the library coda in R (Plummer et al., 2006) which generated a mean phylorate plot with mean and model-averaged diversification rates along every branch of the tree. We also studied if rate shifts exist along individual branches that maximize the marginal probability of change in diversification rate.

Bioclimatic niche analyses

In order to select the relevant bioclimatic variables that most contributed to the variability of the data, we computed a correlation matrix and converted it into a matrix of distances visualized as a dendrogram (Benítez-Benítez et al., 2018), with *stats* implemented in R package. We subsequently selected only one variable per clade when the branch longitude was below 0.5. Then, we also applied the Variance Inflation Factor (VIF) as an estimation

of the multicollinearity effect on model variance, using HH package (Heiberger, 2017). Those variables with VIF>5 were excluded.

We tested the Phylogenetic Niche Conservatism hypothesis between this pairwise comparison group using the function *greater* in *ecospat* package (Di Cola et al., 2017) in order to test if the niche has been preserved or not. We assigned SA clade niche as reference since NZ was colonized from SA (rand.type=2, and thus both niches could not be simultaneously shifted.

Biogeographic analyses

The potential distribution of species can be estimated using ecological niche modelling, combining species occurrence records with environmental data to predict the presence or absence in certain regions (Guisan & Thuiller, 2005; Soberón & Peterson, 2005). We used SDMs to evaluate the potential current distribution of sect. *Junciformes* species in SA in order to establish the biogeographic areas for AAR analyses (Appendix S5). We implemented four different algorithms (MaxEnt, Generalized Linear Models (GLM), Generalized Additive Models (GAM) and Random Forest (RF)) using Biomod2 R package (Thuiller, 2014). We performed the ensemble models for each species (including only those with more than 5 georeferenced occurrences). Finally, we used two methods with a threshold>0.7 to assess metrics for building models: True Skill Statistics (TSS; Allouche et al., 2006) and Area Under the Curve (AUC; Swets, 1988). In addition, biogeographic areas across SA were delimited through current occurrence points with Infomap Bioregions (Edler et al., 2017). We adapted the algorithm based on the partition of geographical space into quadratic grid cells reaching a maximum cell size of 8◦ with 608 species occurrence records. We tested the coding of seven, eight, nine and eleven biogeographic areas for AAR of sect. *Junciformes*.

References

- Allouche, O., Tsoar, A., and Kadmon, R. (2006). Assessing the accuracy of species distribution models: Prevalence, kappa and the true skill statistic (TSS): Assessing the accuracy of distribution models. *Journal of Applied Ecology* 43, 1223-1232. doi: 10.1111/j.1365-2664.2006.01214.x
- Benítez-Benítez, C., Escudero, M., Rodríguez-Sánchez, F., Martín-Bravo, S., and Jiménez-Mejías, P. (2018). Pliocene-Pleistocene ecological niche evolution shapes the phylogeography of a Mediterranean plant group. *Molecular Ecology* 27, 1696–1713. doi: 10.1111/mec.14567
- Blanco-Pastor, J. L., Vargas, P., and Pfeil, B. E. (2012). Coalescent simulations reveal hybridization and Incomplete Lineage Sorting in Mediterranean *Linaria*. *PLoS ONE* 7, e39089. doi: 10.1371/journal.pone.0039089
- Darriba, D., Taboada, G. L., Doallo, R., and Posada, D. (2012). jModelTest 2: More models, new heuristics and parallel computing. *Nature Methods* 9, 772. doi: 10.1038/nmeth.2109
- Di Cola, V., Broennimann, O., Petitpierre, B., Breiner, F. T., D’Amen, M., Randin, C., et al. (2017). ecospat: An R package to support spatial analyses and modeling of species niches and distributions. *Ecography* 40, 774–787. doi: 10.1111/ecog.02671
- Edler, D., Guedes, T., Zizka, A., Rosvall, M., and Antonelli, A. (2016). Infomap Bioregions: Interactive Mapping of Biogeographical Regions from Species Distributions. *Systematic Biology* 66, 197-204. doi: 10.1093/sysbio/syw087
- Guisan, A., and Thuiller, W. (2005). Predicting species distribution: Offering more than simple habitat models. *Ecology Letters* 8, 993–1009. doi: 10.1111/j.1461-0248.2005.00792.x

- Heiberger, R. M. (2017). R package "HH": statistical analysis and data display v. 3.1-34, eds. Heiberger and Holland. https://cran.r-project.org/web/packages/HH/index.html
- Ho, S. Y. W., and Phillips, M. J. (2009). Accounting for Calibration Uncertainty in Phylogenetic Estimation of Evolutionary Divergence Times. *Systematic Biology* 58, 367–380. doi: 10.1093/sysbio/syp035
- Jiménez-Mejías, P., Martinetto, E., Momohara, A., Popova, S., Smith, S. Y., and Roalson, E. H. (2016). A commented synopsis of the Pre-Pleistocene fossil record of *Carex* (Cyperaceae). *The Botanical Review* 82, 258-345. doi: 10.1007/s12229-016-9169-7
- Plummer, M., Best, N., Cowles, K., and Vines, K. (2006). CODA: Convergence diagnosis and output analysis for MCMC. *R News* 6, 7-11. ISSN 1609-3631
- Rabosky, D. L., Grundler, M., Anderson, C., Title, P., Shi, J. J., Brown, J. W., et al. (2014). BAMMtools: An R package for the analysis of evolutionary dynamics on phylogenetic trees. *Methods in Ecology and Evolution* 5, 701–707. doi: 10.1111/2041-210X.12199
- Rambaut, A. (2014). FigTree, tree figure drawing tool v. 1.4.2. http://tree.bio.ed.ac.uk/software/figtree [Accessed December 15, 2020]
- Rambaut, A., Drummond, A. J., Xie, D., Baele, G., and Suchard, M. A. (2018). Posterior summarisation in Bayesian phylogenetics using Tracer 1.7. *Systematic Biology* 67, 901-904. doi: 10.1093/sysbio/syy032
- Soberón, J., and Peterson, A. T. (2005). Interpretation of models of fundamental ecological niches and species' distributional areas. *Biodiversity Informatics, 2,* 1-10. doi: 10.17161/bi.v2i0.4
- Swets, J. (1988). Measuring the accuracy of diagnostic systems. *Science*, 240, 1285–1293. doi: 10.1126/science.3287615
- Thuiller, W. (2014). Editorial commentary on 'BIOMOD - optimizing predictions of species distributions and projecting potential future shifts under global change.' *Global Change Biology* 20, 3591–3592. doi: 10.1111/gcb.12728

SUPPLEMENTARY DATA 2

Phylogenetic results for subg. *Psyllophorae*

The length of the final concatenated matrix, which contained 105 sequences including species belonging to subg. *Psyllophorae* and outgroup species, was 2797 bp. Specifically, the matrices contained 57 samples from sect. *Junciformes,* 21 samples from sect. *Psyllophorae* and 17 from sect. *Schoenoxiphium*. The number of DNA sequences per molecular marker are as follows: 100 for ETS, 95 for ITS, 93 for matK, and 92 for rps16. The best evolutionary model for each molecular marker according to AIC scores as obtained from jModelTest was: SYM+G for ITS and GTR+G for ETS, matK, and rps16. Analyses from BI and ML of each matrix resulted in similar topologies (results not shown). In order to simplify, the results included in Appendix S8 are based on the final matrix including all concatenated markers, as it was the best resolved topology among different obtained trees (Appendix S8). We represented well-supported clades with Posterior Probability (PP) > 0.9 and Bootstrap Support (BS) > 75.

Subgenus *Psyllophorae* was recovered in a strongly supported monophyletic group (PP=1, BS=94) including sects. *Psyllophorae*, *Schoenoxiphium* and *Junciformes.* Similarly, all these sections formed highly supported clades in our phylogeny. Accordingly, the well-supported monophyletic clade constituted by the sect. *Psyllophorae* (Clade A: PP=1, BS=89) was sister to Clade B which contained the current

sects. *Junciformes* and *Schoenoxiphium* (PP=1). The clade containing sect. *Psyllophorae* was in turn subdivided into two main highly supported sister clades: The well-supported *C. distachya*-clade (Clade A1: PP=1; BS=100) and the other highly supported clade constituted by the rest of species belonging to sect. *Psyllophorae* (Clade A2; PP=1; BS=91). In addition, the second diverging clade showed two highly supported and unequivocal sister groups (Clade A2.1: PP=1, BS=100; Clade A2.2: PP=1, BS=91). On the other hand, within Clade B from the Southern Hemisphere (PP=1) were recovered two subclades: (1) South-African sect. *Schoenoxiphium* (Clade B1: PP=0.99, BS=77), and (2) SA and SW Pacific sect. *Junciformes* (Clade B2: PP=1, BS=99). More details related to inner phylogenetic relationships in the sect. *Schoenoxiphium* are found in Luceño et al. (2021). Within sect. *Junciformes* two main well-supported clades were inferred: (i) Aciculares-clade (PP=1), comprising species from NZ (NZ-clade; PP=1, BS=99) and SA (SA-clade; PP=1), and (ii) a poorly supported clade including three monospecific, well-supported lineages for *C. camptoglochin* (PP=1, BS=100), *C. vallis-pulchrae* Phil. (PP=1, BS=100) and *C. phalaroides* (PP=1, BS=100), as well as Junciformes-clade (PP = 1, BS=95), composed of species exclusively from southern SA. Within the latter clade, *C. argentina* Barros was inferred as the earliest diverging lineage sister to the core Junciformes (PP=0.97). In turn, the core Junciformes recovered was subdivided into two subclades: (i) Junciformes Lineage A (PP=1, BS=97), mainly composed of species inhabiting N Patagonia (see Figure 3C), and (ii) Junciformes Lineage B (PP = 1, BS=94) conformed by species from N-S Patagonia (see Figure 2C).
