# Supplementary material for: An Evolutionary Study of Carex Subg. Psyllophorae (Cyperaceae) Sheds Light on a Strikingly Disjunct Distribution in the Southern Hemisphere, With Emphasis on Its Patagonian Diversification
Source: Front Plant Sci. 2021 Nov 8;12:735302. doi: 10.3389/fpls.2021.735302 (PMC8606891; doi:10.3389/fpls.2021.735302)

SUPPLEMENTARY FIGURE 1. Comparison of density plots expressing the frequency of distribution of each uncorrelated selected variable (AMT, TAR, MTDQ, AP, TRI) in those species belonging to *Carex* sect. *Junciformes* within Aciculares-clade, studying independently NZ and SA sister clades.

Aciculares-clade NZ

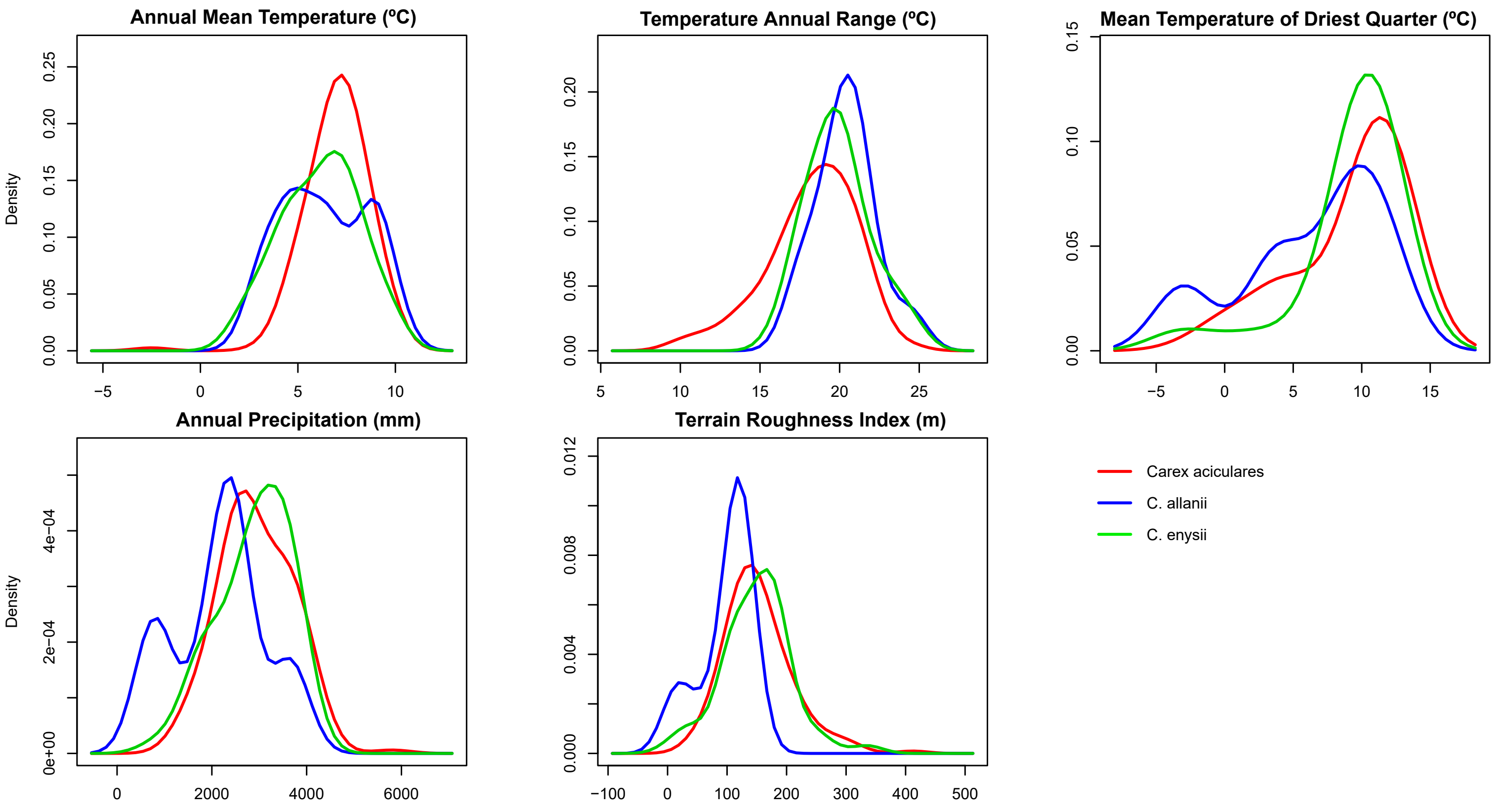

Aciculares-clade SA

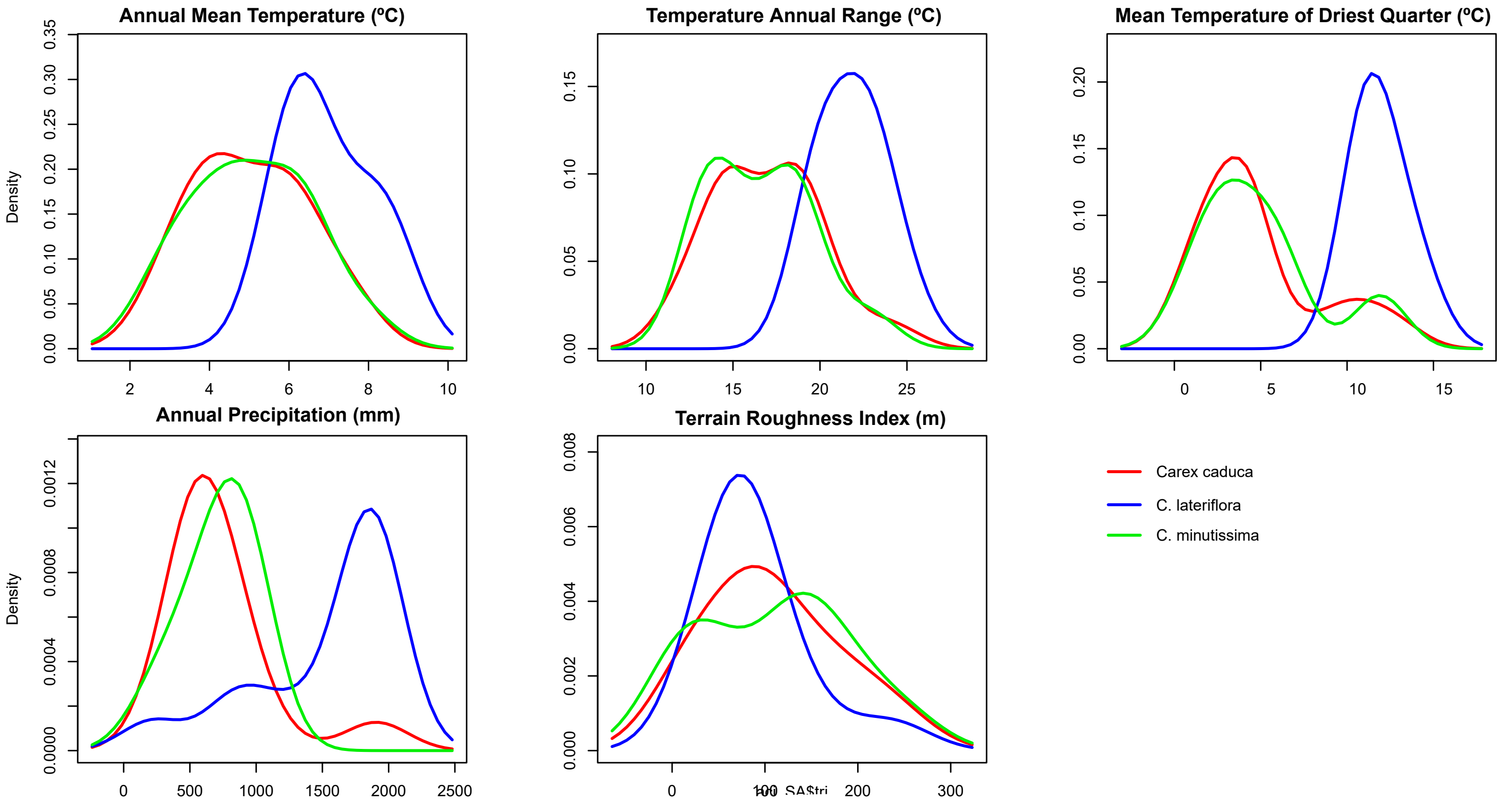

SUPPLEMENTARY FIGURE 2. Comparison of density plots expressing the frequency of distribution of each uncorrelated selected variable (AMT, TAR, MTDQ, AP, TRI) in those species belonging to *Carex* sect. *Junciformes* within Junciformes-clade, studying independently Lineage A and Lineage B sister clades.

Junciformes-clade Lineage A

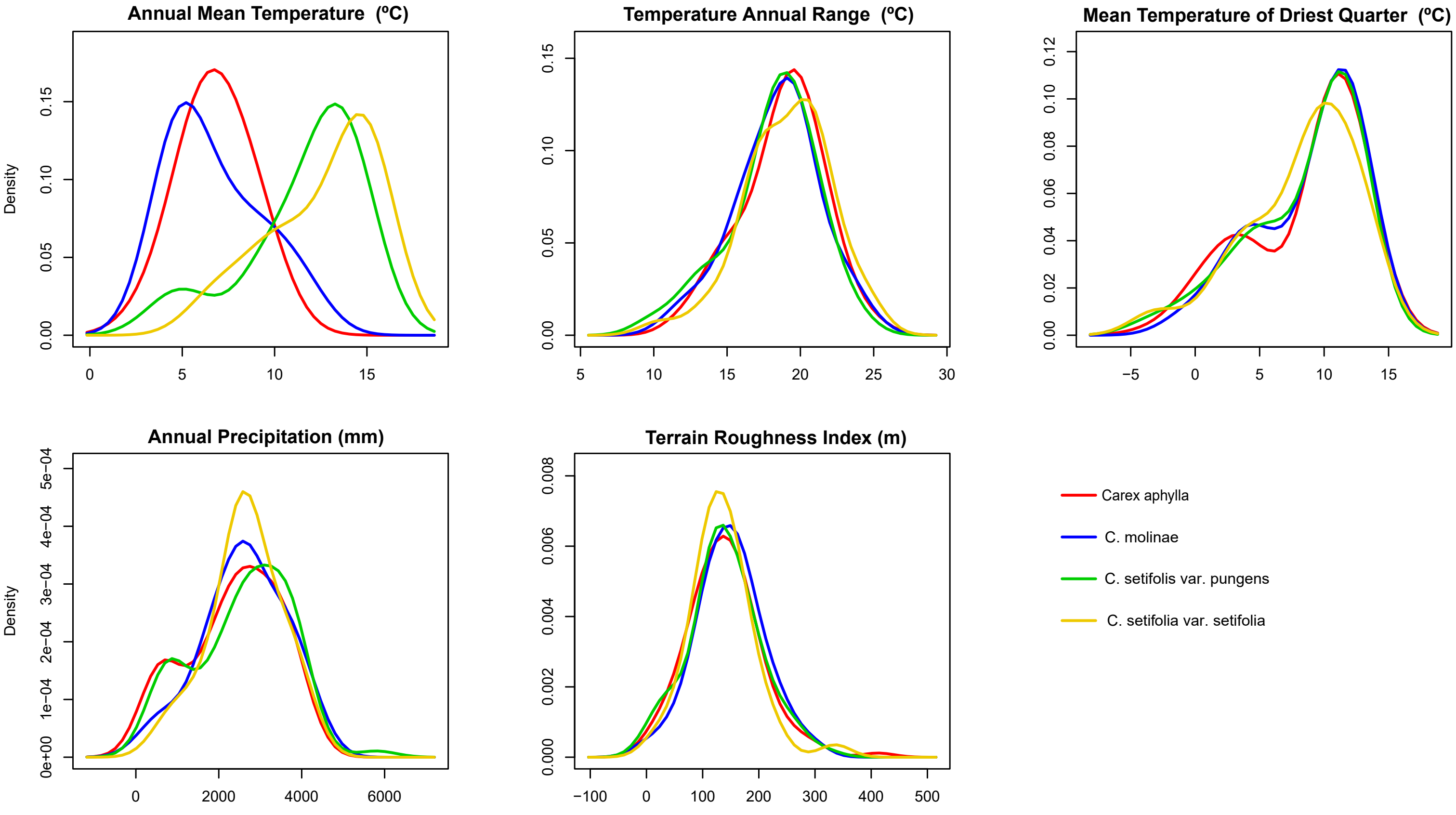

Junciformes-clade Lineage B

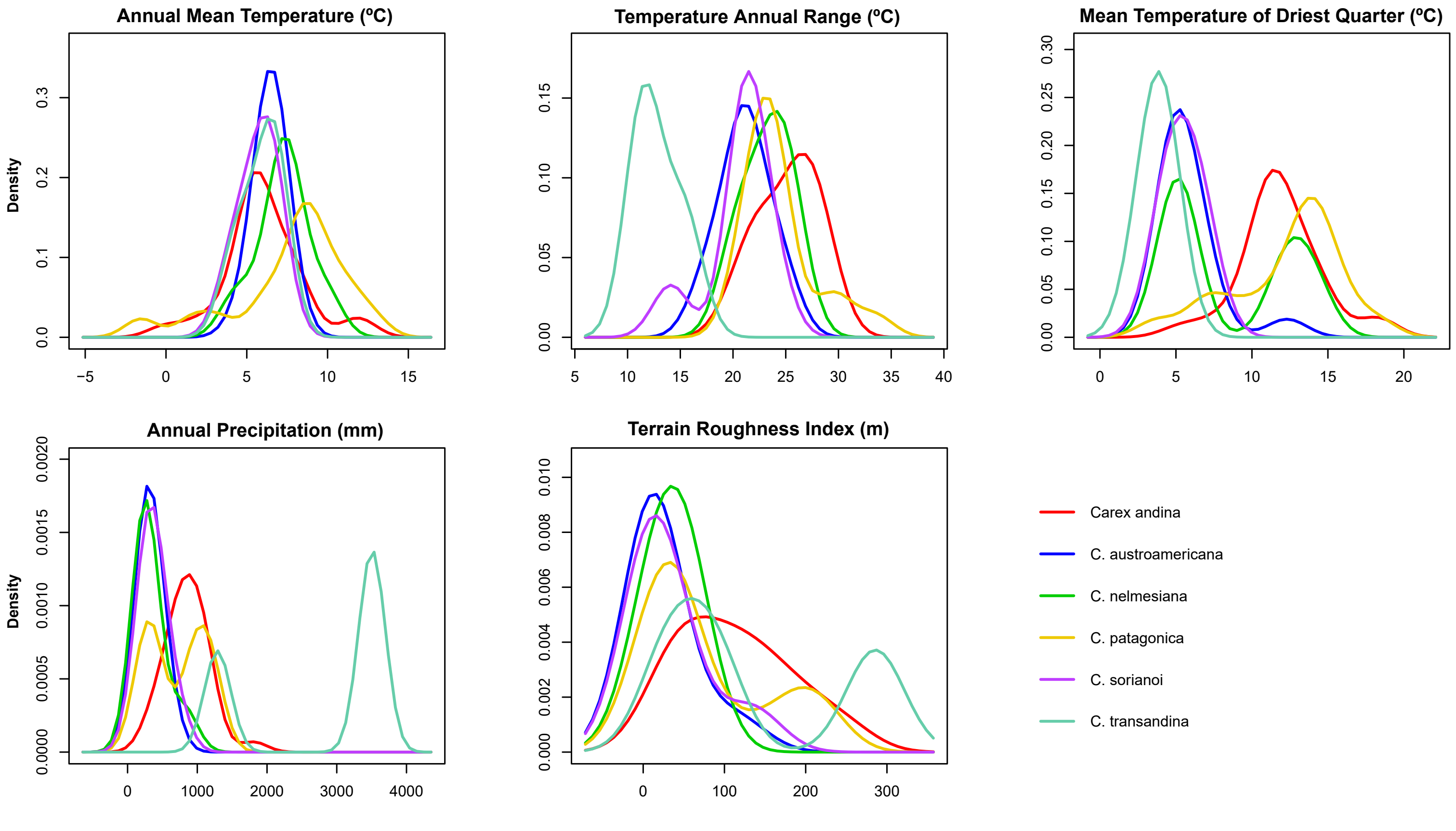

SUPPLEMENTARY FIGURE 3. Distribution maps of species occurrences of *Carex* sect. *Junciformes*, which have been used for the biogeographic and bioclimatic analyses.

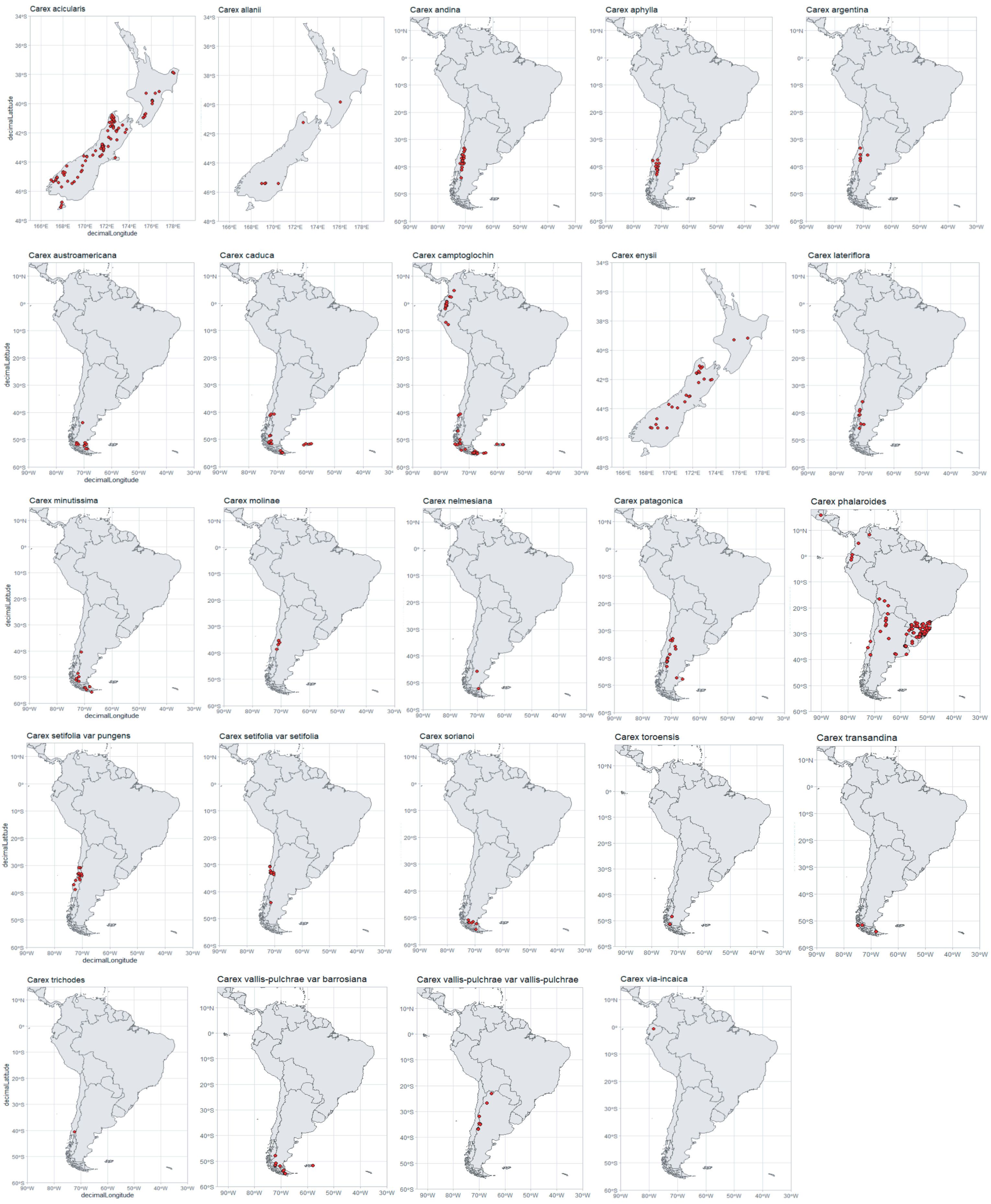

SUPPLEMENTARY FIGURE 4. Species distribution modelling of the current potential distribution of South American species belonging to *Carex* sect. *Junciformes* with more than five number of populations using Biomod2 package in R.

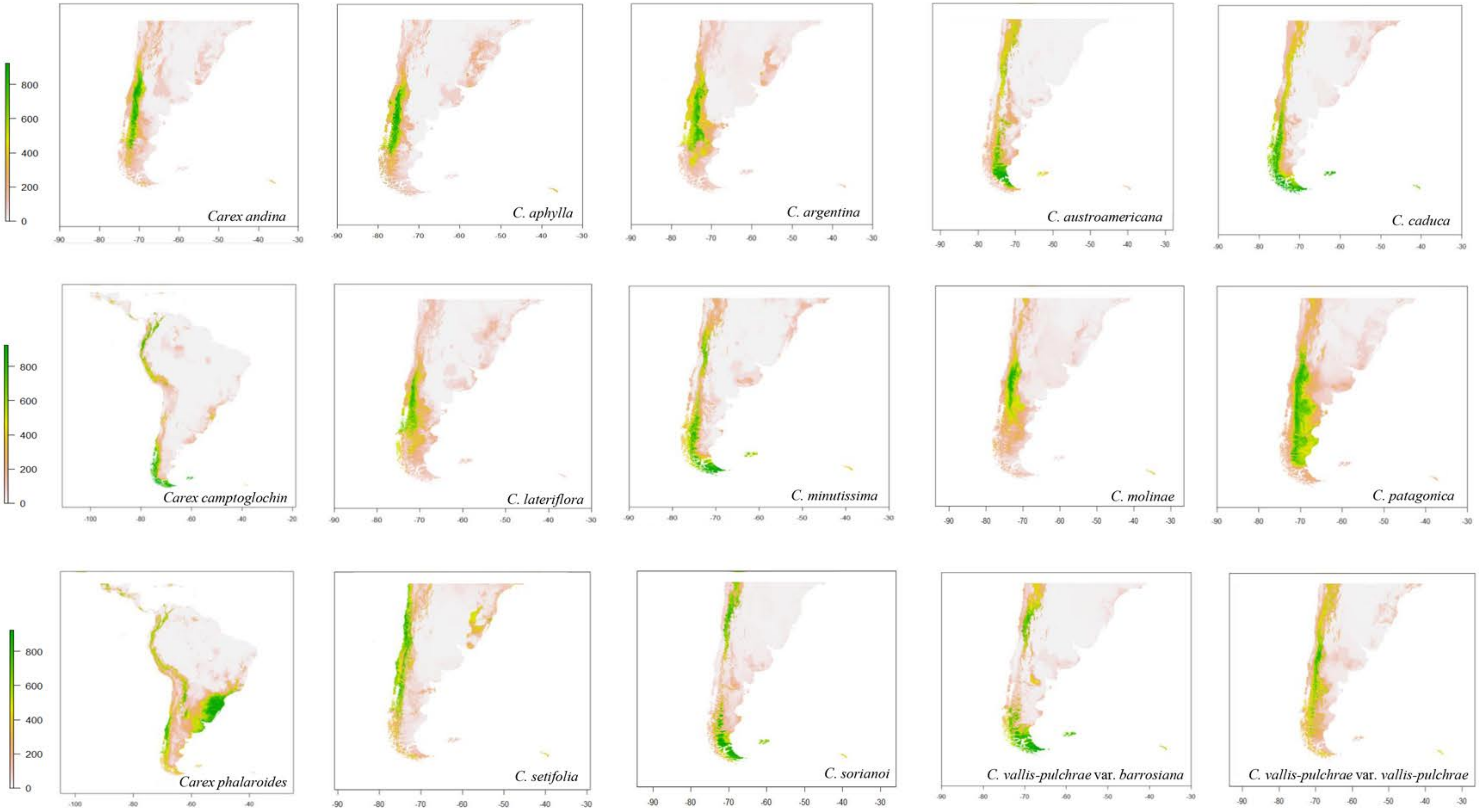

SUPPLEMENTARY FIGURE 5. Bayesian phylogenetic tree obtained from the concatenation of nrDNA (ITS and ETS) and ptDNA (matK and rps16) sequences of *Carex* subg. *Psyllophorae* including multiple tips per taxa. Numbers above branches correspond to the posterior probability (PP > 0.9), and those below branches to Bootstrap support (BS > 75).

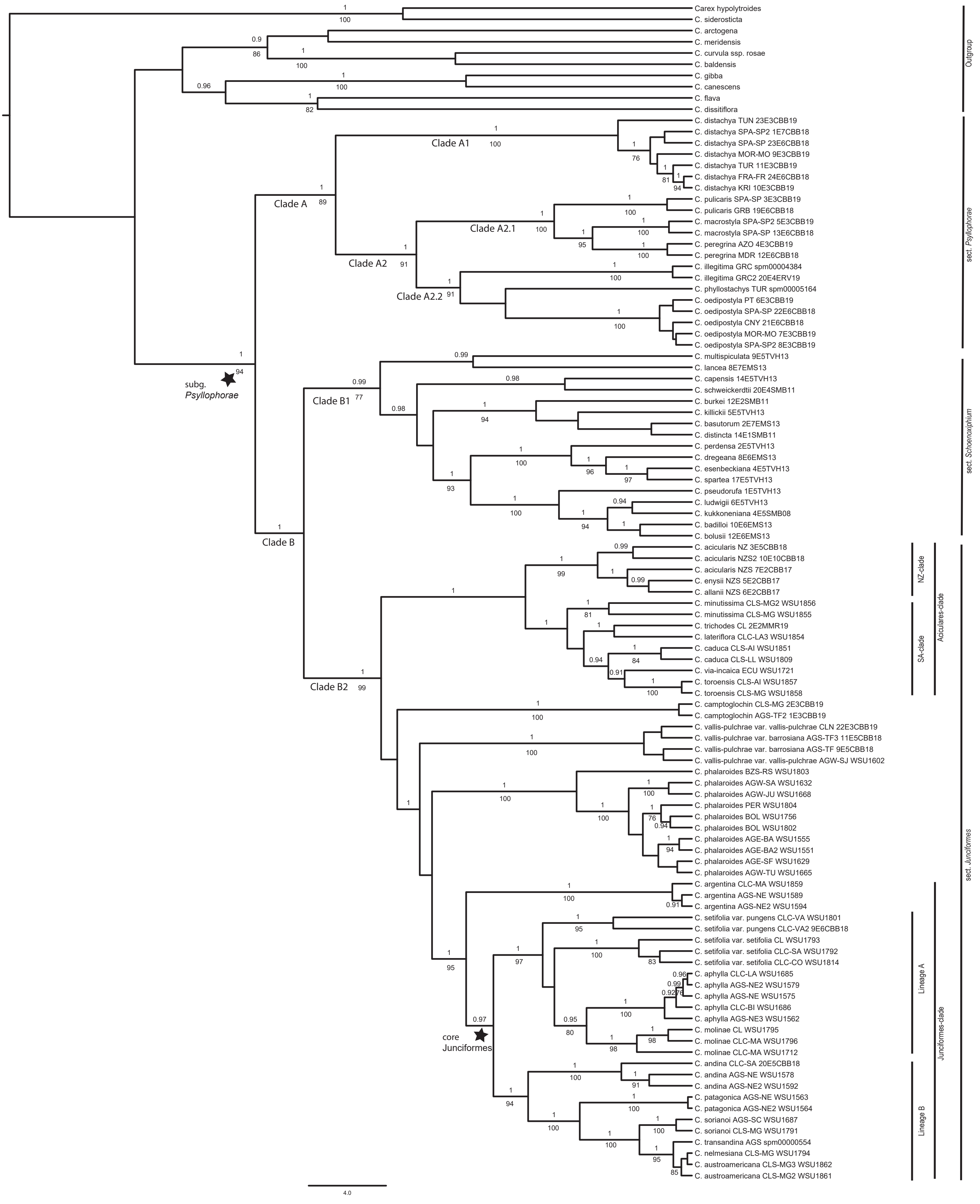

SUPPLEMENTARY FIGURE 6. Chronogram based on the phylogenetic tree of *Carex* subg. *Psyllophorae* using BEAST. Three fossils were placed at deep nodes as primary calibration points, as well as a secondary calibration point for the crown node of the subgenus. Ages are indicated at nodes. Tip labels represent the same accessions included in Supplementary Figure 5.

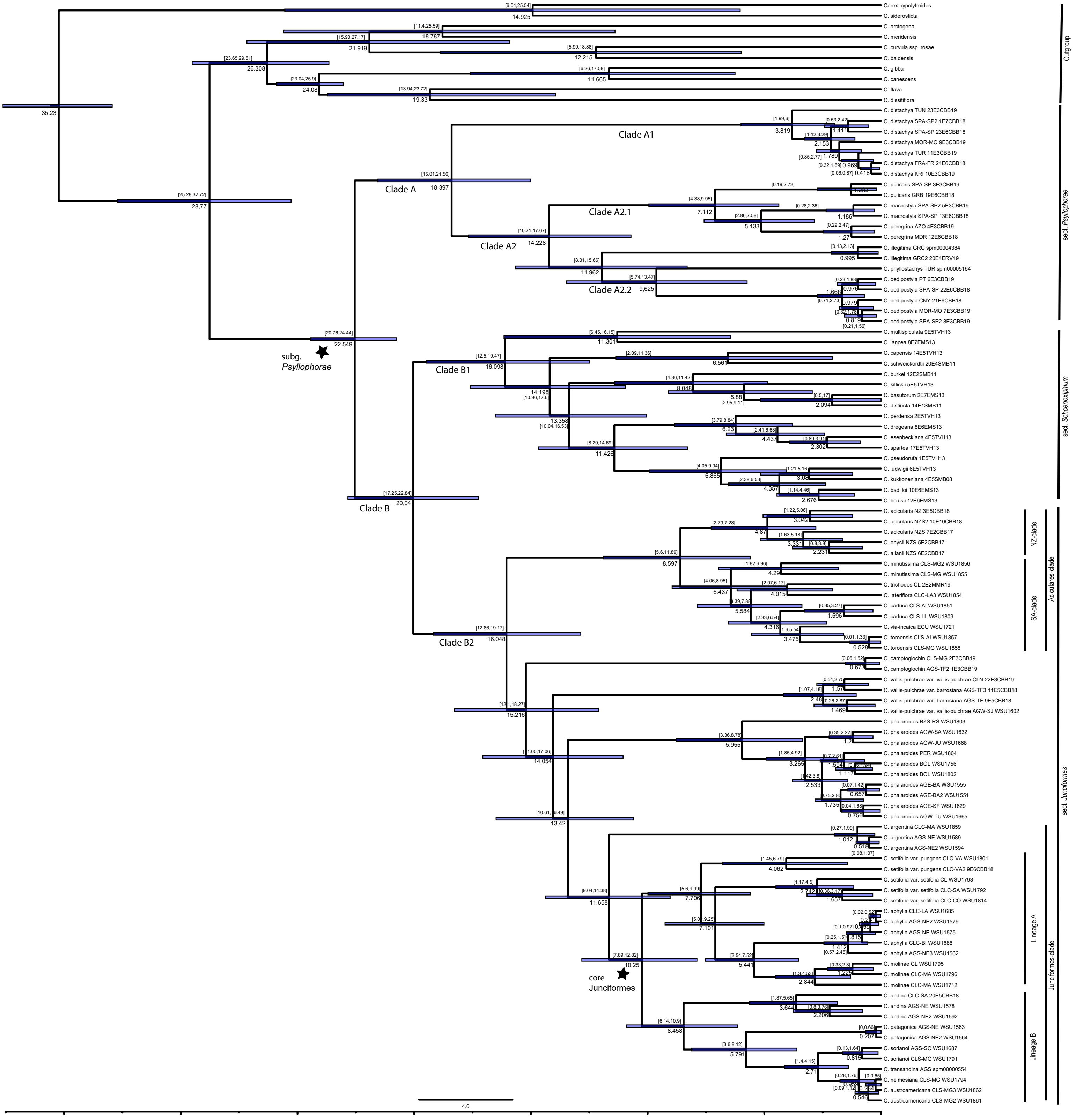

SUPPLEMENTARY FIGURE 7. Principal Component Analysis displaying e-space for species belonging to *Carex* sect. *Junciformes*, representing mean values of uncorrelated bioclimatic variables used for the niche evolution analyses.

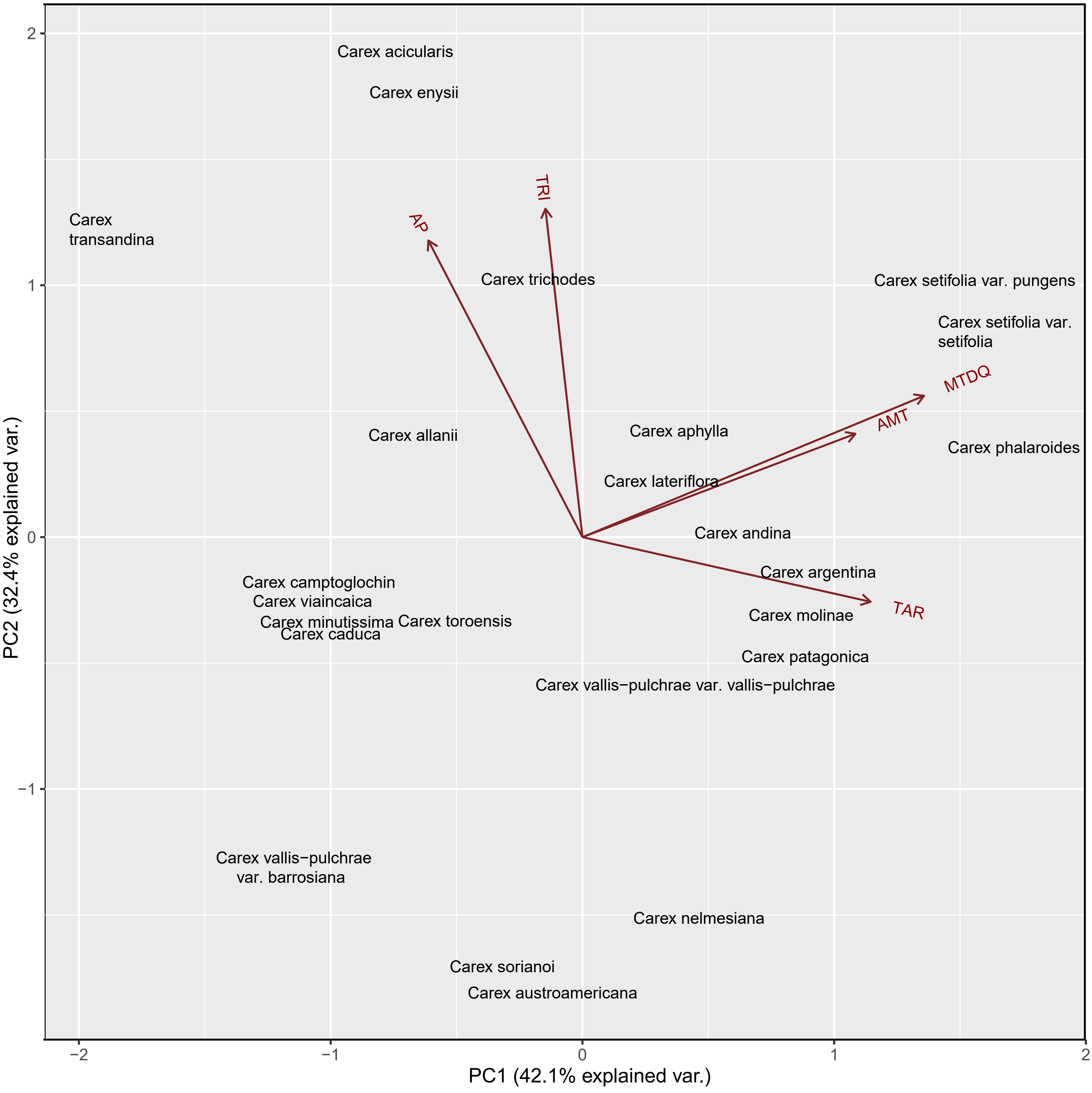

SUPPLEMENTARY FIGURE 8. Niche overplot performing an e-space pairwise comparison between two clades (SA vs. NZ) belonging to *Carex* sect. *Junciformes* within Aciculares-clade. Principal Component Analysis Mean used mean values for the five selected variables (AMT, TAR, MTDQ, AP, TRI), whose contributions were retrieved by PC1 (41.11%) and PC2 (25.87%) in both axes. SA-clade is shown in blue color and NZ-clade in orange.

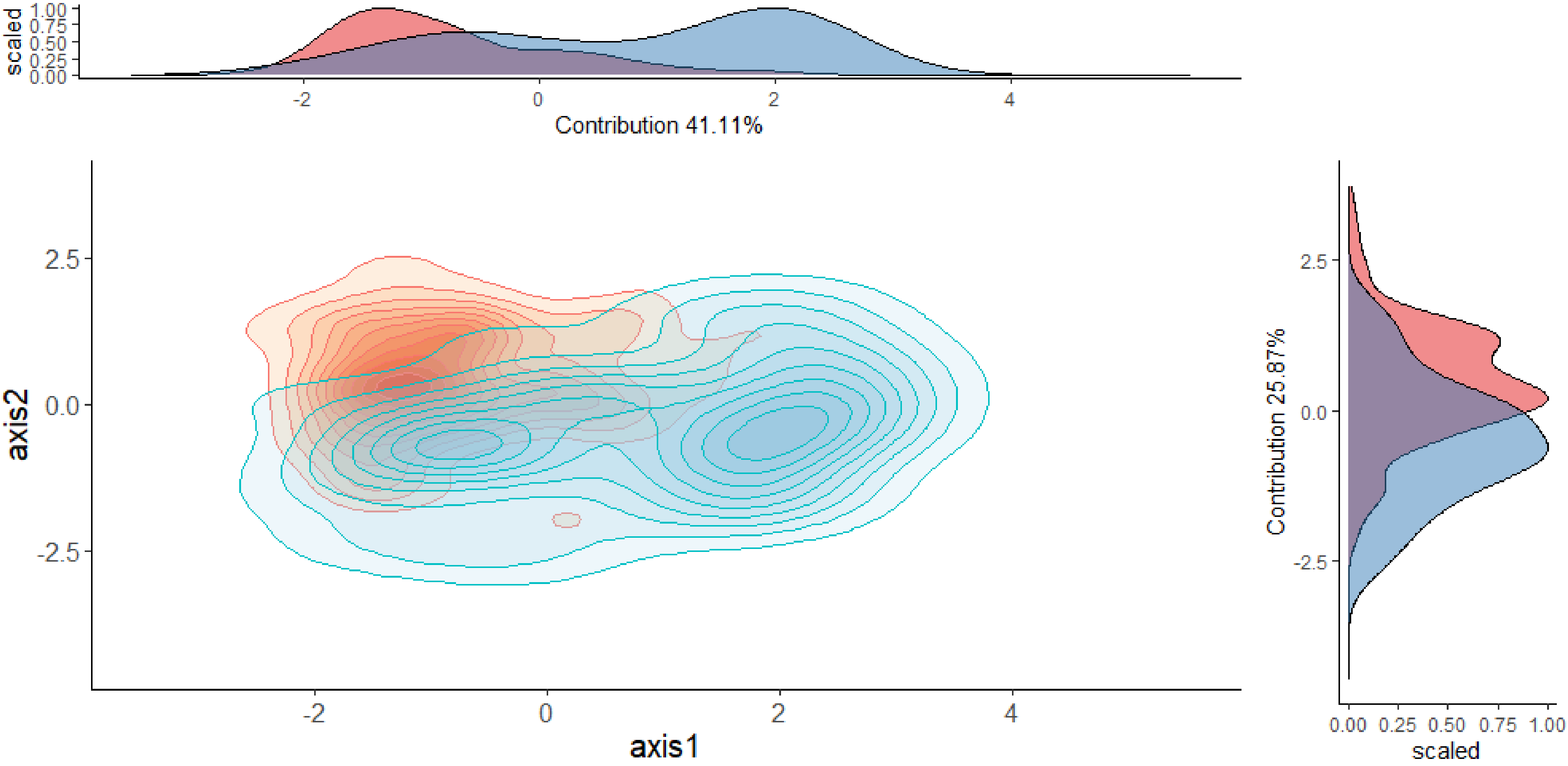

SUPPLEMENTARY FIGURE 9. Ancestral area reconstruction based on calibrated tree of *Carex* subg. *Psyllophorae* under DEC model as implemented in BioGeoBEARS. The tree displays the most probable area or combination of areas on each node with colored squares. Colors of areas correspond to Figure 3A. Additional colors are combinations of areas.

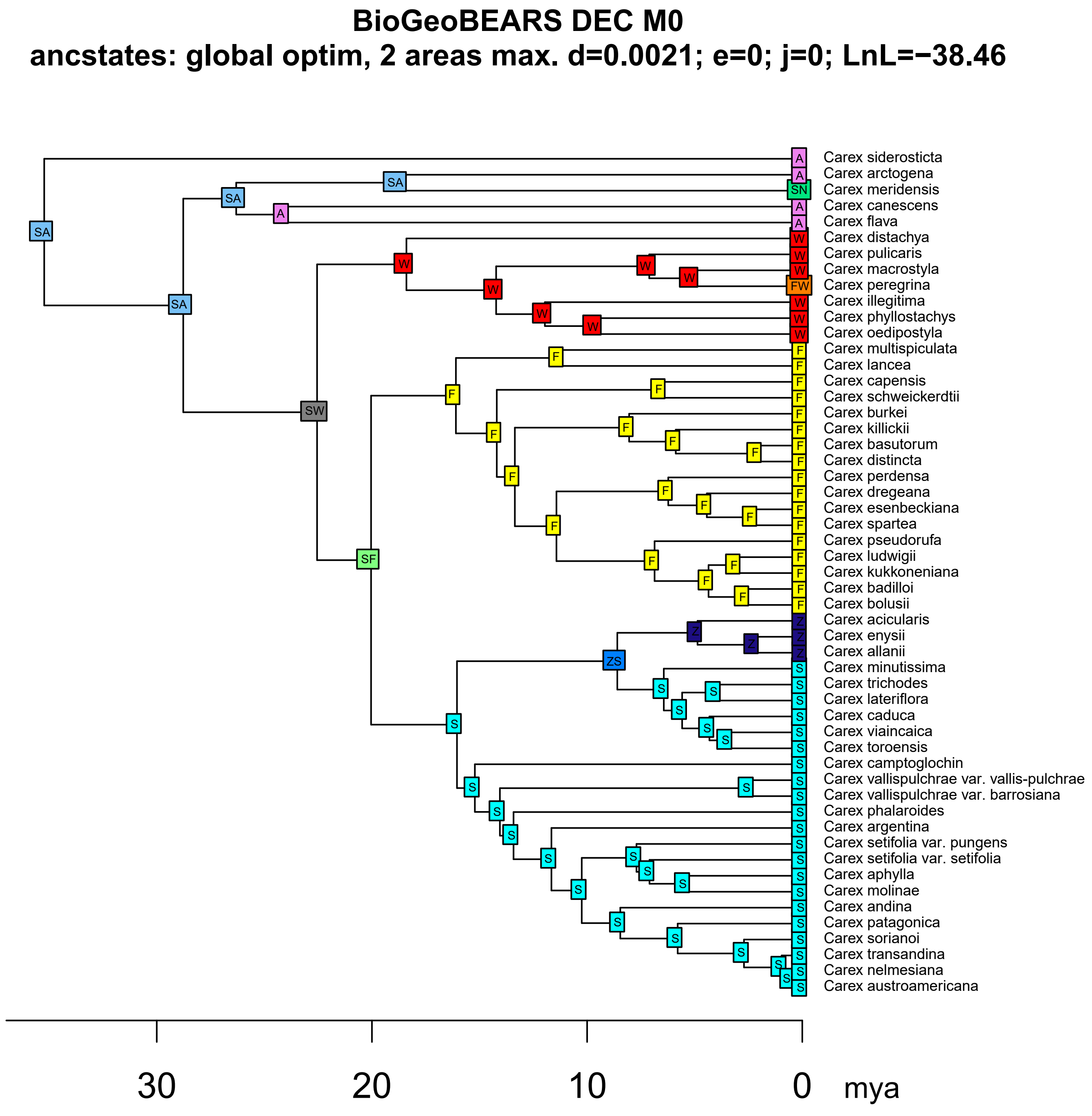

SUPPLEMENTARY FIGURE 10. Ancestral area reconstruction based on calibrated tree of *Carex* subg. *Psyllophorae* under BAYAREA-like model as implemented in BioGeoBEARS. The tree displays the most probable area or combination of areas on each node with colored squares. Colors of areas correspond to Figure 3A. Additional colors are combinations of areas.

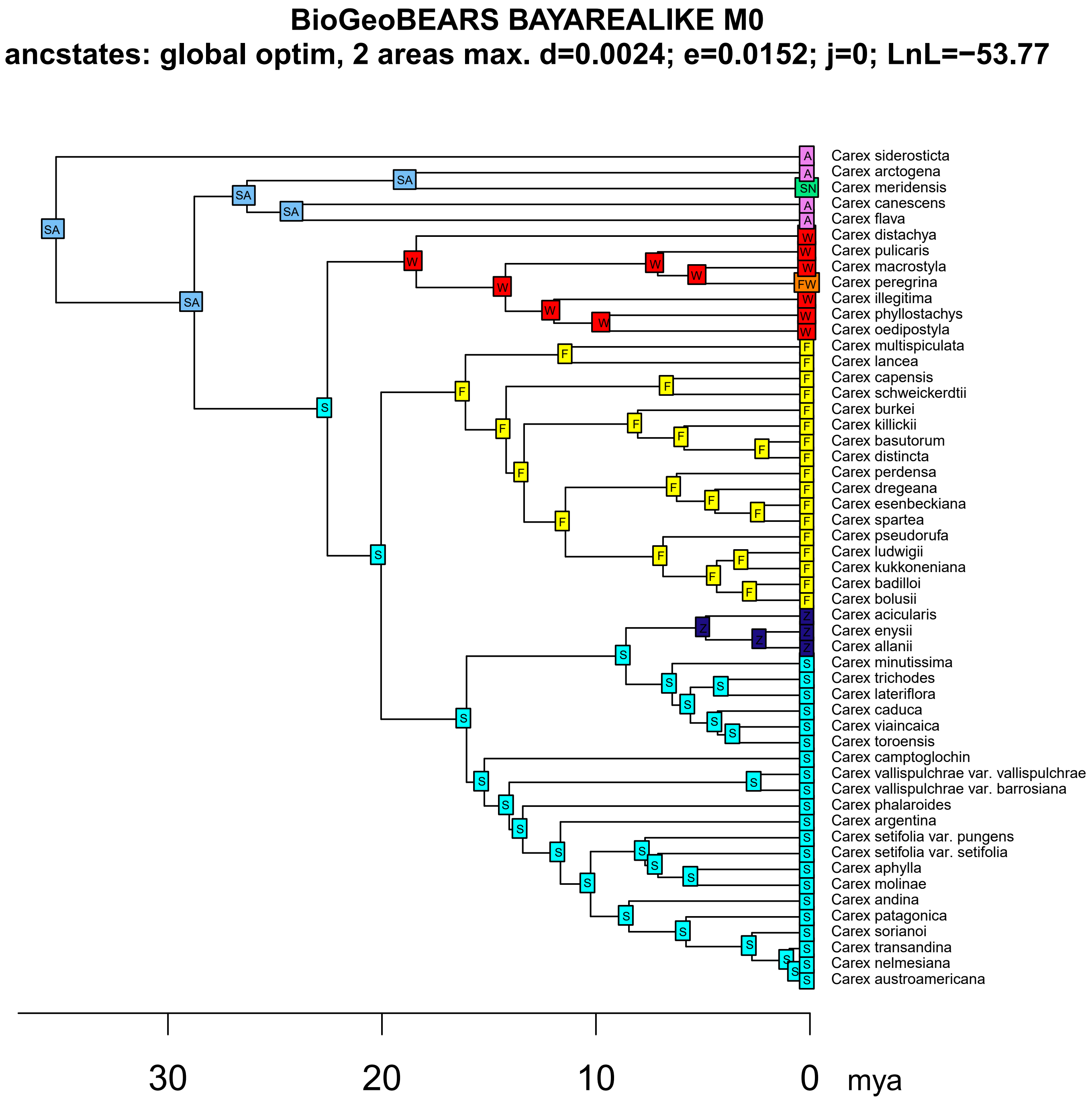

SUPPLEMENTARY FIGURE 11. Ancestral area reconstruction based on pruned calibrated tree of *Carex* sect. *Junciformes* under DIVA-like model as implemented in BioGeoBEARS. The tree displays the most probable area or combination of areas on each node with colored squares. Colors of areas correspond to Figure 3C. Additional colors are combinations of areas.

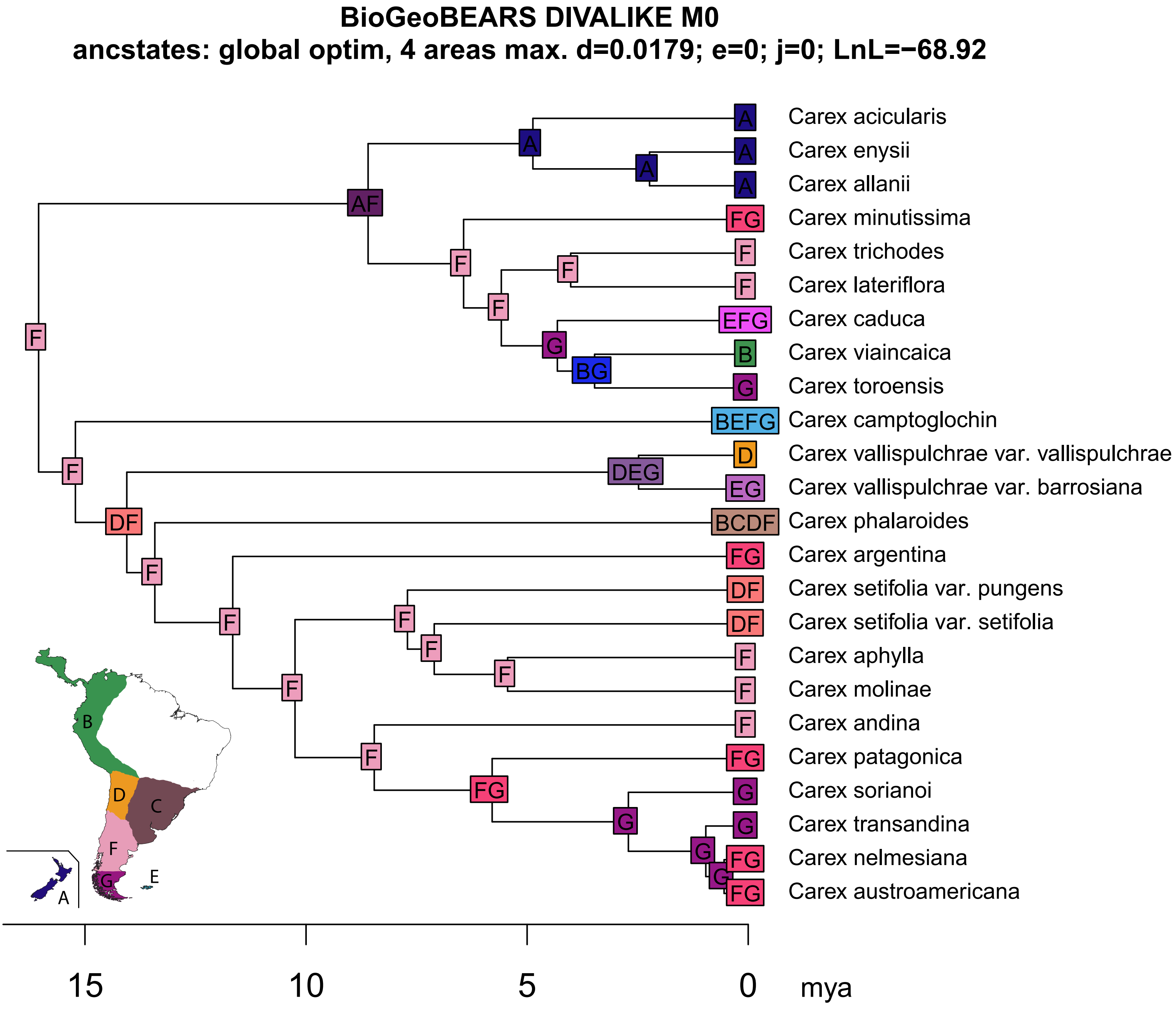

SUPPLEMENTARY FIGURE 12. Ancestral area reconstruction based on pruned calibrated tree of *Carex* sect. *Junciformes* under BAYAREA-like model as implemented in BioGeoBEARS. The tree displays the most probable area or combination of areas on each node with colored squares. Colors of areas correspond to Figure 3C. Additional colors are combinations of areas.

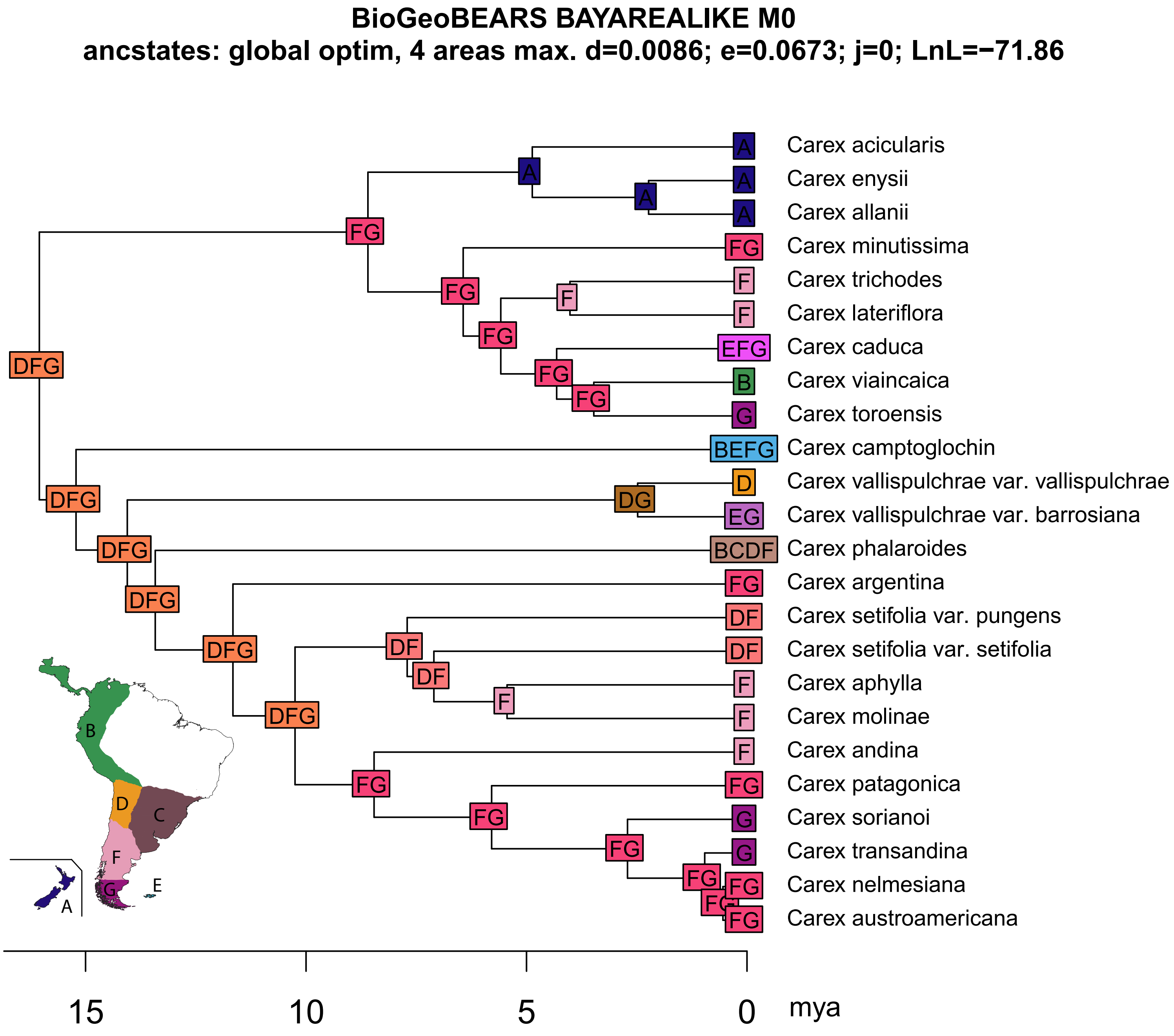

Supplement: Supplementary file 1 [file Data_Sheet_1.zip › Supplementary Figures.PDF]
