# Supplementary material for: An Evolutionary Study of Carex Subg. Psyllophorae (Cyperaceae) Sheds Light on a Strikingly Disjunct Distribution in the Southern Hemisphere, With Emphasis on Its Patagonian Diversification
Source: Front Plant Sci. 2021 Nov 8;12:735302. doi: 10.3389/fpls.2021.735302 (PMC8606891; doi:10.3389/fpls.2021.735302)
Supplement: Supplementary file 1 [file Data_Sheet_1.zip › Supplementary Table 1.PDF]

SUPPLEMENTARY TABLE 1. List of material of taxa sampled. Specimens sequenced in the present study are marked with an asterisk (*).

Taxa	Country	Locality	Sample code	Voucher (Herbarium)	ETS	ITS	*matK*	*rps16*
Sect. ***Junciformes*** (Boeck.) Kük.								
Carex acicularis Boott	New Zealand	New Zealand, Canterbury Land District, Mackenzie Basin, Fork	NZ 3E5CBB18	K. Lloyd (CHR624240)	MZ892124*	MZ892197*	MZ891993*	-
		New Zealand, Southland Land District Fiordland, Saddle Hill	NZS 7E2CBB17	B. Rance s.n. (CHR638461)	MZ892126*	MZ892199*	MZ891995*	MZ892058*
		New Zealand, Southland Land District, Eyre Mtn.	NZS2 10E10CBB18	B. Rance, KF 780/18	MZ892125*	MZ892198*	MZ891994*	MZ892057*
Carex allanii Hamlin	S. New Zealand	New Zealand, Canterbury Land District, Maitland	NZS 6E2CBB17	K. A. Ford & A. Shanks, s.n. (CHR619158)	MZ892127*	MZ892200*	MZ891996*	MZ892059*
Carex andina Phil.	C. Chile to SW. Argentina	Chile, Santiago, Nevado Valley	CLC-SA 20E5CBB18	M. Luceño & R. Álvarez (UPOS1837)	MZ892128*	MZ892201*	MZ891997*	MZ892060*
		Argentina, Neuquen	AGS-NE WSU1578	S. Martín-Bravo, P. Jiménez-Mejías & G.E. Rodríguez-Palacios 54SMB15 (UPOS)	MZ892140*	MZ892213*	MZ892008*	MZ892071*
		Argentina, Neuquen	AGS-NE2 WSU1592	S. Martín-Bravo, P. Jiménez-Mejías & G.E. Rodríguez-Palacios 68SMB15 (UPOS)	MZ892139*	MZ892212*	MZ892007*	MZ892070*
Carex aphylla Kunth	Chile, S. Argentina	Argentina, Neuquen	AGS-NE WSU1575	S. Martín-Bravo, P. Jiménez-Mejías & G.E. Rodríguez-Palacios 50SMB15 (UPOS)	MZ892131*	MZ892204*	MZ892000*	MZ892063*
		Argentina, Neuquen	AGS-NE2 WSU1579	S. Martín-Bravo, P. Jiménez-Mejías & G.E. Rodríguez-Palacios 55SMB15 (UPOS)	MZ892129*	MZ892202*	MZ891998*	MZ892061*
		Chile, Cautin Province	CLC-LA WSU1685	K. H. W. Rechinger (M-MSB 0223515)	MZ892133*	MZ892206*	MZ892001*	MZ892064*
		Chile, Biobio Province	CLC_BI WSU1686	K.H.W. Rechinger (M-MSB 63904)	MZ892132*	MZ892205*	-	-
		Argentina, Neuquen	AGS-NE3 WSU1562	S. Martín-Bravo, P. Jiménez-Mejías, G.E. Rodríguez-Palacios & M. Waterway 35SMB15 (UPOS)	MZ892130*	MZ892203*	MZ891999*	MZ892062*

Carex argentina Barros	W. Argentina	Argentina, Neuquen	AGS-NE WSU1589	S. Martín-Bravo, P. Jiménez-Mejías & G.E. Rodríguez-Palacios 65SMB15 (UPOS)	MZ892135*	MZ892208*	MZ892003*	MZ892066*
		Argentina, Neuquen	AGS-NE2 WSU1594	S. Martín-Bravo, P. Jiménez-Mejías, G.E. Rodríguez-Palacios 70SMB15 (UPOS)	MZ892134*	MZ892207*	MZ892002*	MZ892065*
		Chile, Talca Province	CLC-MA WSU1859	N. Garcia & T. Villasenor (CONC3908)	MZ892136*	MZ892209*	MZ892004*	MZ892067*
Carex austroamericana G.A. Wheeler	S. Chile a S. Argentina	Chile, Magallanes Province	CLS-MG2 WSU1861	E. Dominguez (CONC177)	MZ892137*	MZ892210*	MZ892005*	MZ892068*
		Chile, Ultima Esperanza Province	CLS-MG3 WSU1862	E. Dominguez (CONC347)	MZ892138*	MZ892211*	MZ892006*	MZ892069*
Carex caduca Boott	S. Chile, S. Argentina, Falkland Isl.	Chile, Osoruo	CLS-LL WSU1809	G. A. Wheeler (BR7762)	MZ892143*	MZ892216*	MZ892010*	MZ892074*
		Chile, Capitan Prat Province	CLS-AI WSU1851	N. García (CONC63)	MZ892142*	MZ892215*	MZ892009*	MZ892073*
Carex camptoglochin V.I.Krecz.	Colombia to Ecuador, S. Chile to Falkland Isl.	Argentina, Tierra del Fuego	AGS-TF2 1E3CBB19	J. Starr, T. Villaverde, M. Luceño, S. Martín-Bravo & P. Vargas (UPOS3920)	MZ892144*	MZ892217*	MZ892011*	MZ892075*
		Chile, Magallanes-Antarctica	CLS-MG 2E3CBB19	M. Luceño & R. Álvarez (UPOS1814)	MZ892145*	MZ892218*	MZ892012*	MZ892076*
Carex enysii Petrie	New Zealand	New Zealand, Canterbury Land District, Craigieburn Range, Mt Cockayne	NZS 5E2CBB17	K. A. Ford & A. Shanks, KF502/17 (CHR638464)	MZ892153*	MZ892226*	MZ892019*	MZ892084*
Carex lateriflora Phil.	SC. & S. Chile, S. Argentina	Chile, Volcan Province	CLC-LA3 WSU1854	L. Raming (CONC43598)	MZ892158*	-	MZ892023*	MZ892089*
Carex minutissima Barros	S. Chile a SW. Argentina	Chile, Tierra de Fuego	CLS-MG WSU1855	Pisano et al. (CONC8314)	MZ892162*	MZ892233*	MZ892026*	MZ892092*
		Chile, Tierra de Fuego	CLS-MG2 WSU1856	Pisano et al. (CONC8244)	MZ892161*	MZ892232*	MZ892025*	-
Carex molinae Phil.	C. Chile	Chile, El Picago	CL WSU1795	E. Barros (A461)	MZ892163*	MZ892234*	MZ944883*	MZ892093*
		Chile, Curico Province	CLC-MA WSU1796	E. Werderman (A519)	MZ892164*	MZ892235*	MZ892027*	MZ892094*
		Chile, Talca Province	CLC-MA WSU1712	M.F. Garner et al. (E4556)	MZ892141*	MZ892214*	-	MZ892072*
Carex nelmesiana Barros	S. Argentina	Patagonia, Escorial de Pali Aike, Cerro Diablo	CLS-MG WSU1794	E. Pisano (A5205)	MZ892165*	-	MZ892028*	MZ892095*

Carex patagonica Speg.	WC. & SW. Argentina, C. & S. Chile	Argentina, Neuquen	AGS-NE WSU1563	S. Martín-Bravo, P. Jiménez-Mejías, G.E. Rodríguez-Palacios & M. Waterway 36SMB15 (UPOS)	MZ892172*	MZ892241*	MZ892035*	MZ892102*
		Argentina, Neuquen	AGS-NE2 WSU1564	S. Martín-Bravo, P. Jiménez-Mejías & G.E. Rodríguez-Palacios 37SMB15 (UPOS)	MZ892171*	MZ892240*	MZ892034*	MZ892101*
Carex phalaroides Kunth.	Guatemala, NW. Venezuela to Uruguay, Brasil to NE. Argentina to Bolivia, Peru	Argentina, Salta	AGW-SA WSU1632	G. Rodríguez-Palacios, M. Fabbroni & P. Jiménez-Mejías 23GERP15	MZ892179*	MZ892247*	MZ892042*	MZ892109*
		Argentina, Tucuman	AGW-TU WSU1665	G. Rodríguez-Palacios & P. Jiménez-Mejías 70bisGERP15	MZ892180*	MZ892248*	MZ892043*	MZ892110*
		Bolivia, Prov. Florida Samaipata region	BOL WSU1802	C.E. Hinchliff (WS762)	MZ892181*	MZ892249*	MZ892044*	MZ892111*
		Argentina, San Lorenzo	AGE-SF WSU1629	G. Rodríguez-Palacios & P. Jiménez-Mejías 16GERP15	MZ892177*	MZ892245*	MZ892040*	MZ892107*
		Argentina, Lagunas de Yala	AGW-JU WSU1668	G. Rodríguez-Palacios & P. Jiménez-Mejías 80GERP15	MZ892178*	MZ892246*	MZ892041*	MZ892108*
		Bolivia, Chuquisaca	PAR_BOL WSU1756	J. R. I. Wood (NY10845)	MZ892175*	MZ892243*	MZ892038*	MZ892105*
		Brasil, Sao Jose dos Ausentes	PAR_BZS-RS WSU1803	R. Trevisan (WS878)	MZ892176*	MZ892244*	MZ892039*	MZ892106*
		Perú, Puno	PER WSU1804	E. Roalson (WS1449)	MZ892156*	MZ892228*	MZ892022*	MZ892087*
		Argentina, Buenos Aires	AGE-BA WSU1555	S. Martín-Bravo, P. Jiménez-Mejías & G.E. Rodríguez-Palacios 20SMB15 (UPOS)	MZ892155*	MZ892227*	MZ892021*	MZ892086*
		Argentina, Buenos Aires	AGE-BA2 WSU1551	S. Martín-Bravo, P. Jiménez-Mejías & G.E. Rodríguez-Palacios 9SMB15 (UPOS)	MZ892154*	-	MZ892020*	MZ892085*
Carex setifolia var. *pungens* (Boeckeler) Kük.	Chile	Chile, Aconcagua	CLC-VA WSU1801	W.A. Weber & B. Johnston (WS1030)	MZ892188*	-	MZ892050*	MZ892115*

		Chile, Aconcagua	CLC-VA2 9E6CBB18	Gunckel (SI14544)	MZ892187*	-	-	-
Carex setifolia var. *setifolia* Kunze	Peru to Chile	Chile, Santiago	CLC-SA WSU1792	L. Looner (A955)	MZ892186*	-	MZ892049*	-
		Chile	CL WSU1793	E. Barros (A462)	MZ892184*	MZ892251*	MZ892047*	MZ892113*
		Chile, Coquimbo	CLC-CO WSU1814	G.A. Wheeler (BR6158)	MZ892185*	MZ892252*	MZ892048*	MZ892114*
Carex sorianoi Barros	S. Chile to S. Argentina	Argentina, Sta. Cruz	AGS-SC WSU1687	P. Seibert (M-MSB 2133)	MZ892189*	MZ892253*	-	-
		Patagonia, Sierra Aguales	CLS-MG WSU1791	E. Pisano & R. Cardenas (A4737)	MZ892190*	MZ892254*	MZ892051*	MZ892116*
Carex toroensis G.A.Wheeler	S. Chile	Chile, Capitan Prat Province	CLS-AI WSU1857	N. Garcia (CONC78)	MZ892191*	MZ892255*	MZ892052*	MZ892117*
		Patagonia, Fiord Parry	CLS-MG WSU1858	E. Pisano et al. (CONC8812)	-	MZ892256*	MZ892053*	MZ892118*
Carex transandina G.A.Wheeler	S. Chile	Argentina, Tierra del Fuego	AGS spm000554	S. Martín-Bravo et al. 47SMB10 (UPOS4280)	MN759896	MN762540	MN763774	-
Carex trichodes Steud.	S. Chile	Chile	CL 2E2MMR19	D. Penneckamp 2018	-	MZ892257*	MZ944884*	MZ892119*
Carex vallis-pulchrae var. *barrosiana* G.A. Wheeler	S. Chile to Falkland Isl.	Argentina, Tierra del Fuego	AGS-TF 9E5CBB18	S. Laegaard (MO4008221)	MZ892193*	MZ892259*	-	-
		Argentina, Tierra del Fuego	AGS-TF3 11E5CBB18	Perez Haase s/n (SI)	MZ892192*	MZ892258*	-	MZ892120*
Carex vallis-pulchrae var. *vallis-pulchrae* Phil.	Bolivia (Oruro), C. Chile to W. Argentina	Argentina, San Juan	AGW-SJ WSU1602	S. Martín-Bravo, P. Jiménez-Mejías & G.E. Rodríguez-Palacios 80SMB15 (UPOS4253)	MZ892194*	-	MZ892054*	MZ892121*
		Chile, Huasco Province	CLN 22E3CBB19	Yano 55235	MZ892195*	MZ892260*	MZ892055*	MZ892122*
Carex via-incaica Jim.Mejías & Roalson	Ecuador	Ecuador, Cotopaxi National Park	ECU WSU1721	H. Balsley et al. (NY3360)	MZ892196*	MZ892261*	MZ892056*	MZ892123*

Sect. ***Psyllophorae*** Degl.								
Carex distachya Desf.	Mediterranean region	France, Languedoc-Rosellón	FRA-FR 24E6CBB18	S. Martín-Bravo (UPOS8583)	MZ892146*	MZ892219*	MZ892013*	MZ892077*
		Greece, Creta, Kissamos-Koutsomatadas	KRI_10E3CBB19	S. Martín-Bravo & M. Luceño (UPOS264)	MZ892147*	MZ892220*	MZ944880*	MZ892078*
		Morocco, Tanger-Tetuán	MOR-MO 9E3CBB19	P. Jiménez-Mejías, E. Narbona, A.J. Chaparro & M. Parra (UPOS1550)	MZ892148*	MZ892221*	MZ892014*	MZ892079*
		Spain, Toledo	SPA-SP_23E6CBB18	P. Jiménez-Mejías, J.C. Zamora, V. Ferrero, I. Villa & J.E. Rodríguez (UPOS5486)	MZ892150*	MZ892223*	MZ892016*	MZ892081*
		Spain, Barcelona	SPA-SP2 1E7CBB18	P. Jiménez-Mejías, S. Martín-Bravo, E. Maguilla & M. Luceño (UPOS5190)	MZ892149*	MZ892222*	MZ892015*	MZ892080*
		Tunisia	TUN 23E3CBB19	P. Jiménez-Mejías 141PJM13	MZ892151*	MZ892224*	MZ892017*	MZ892082*
		Turkey, Manisa, Borlu	TUR 11E3CBB19	M. Escudero, M. Luceño, S. Martín-Bravo & P. Vargas (UPOS2266)	MZ892152*	MZ892225*	MZ892018*	MZ892083*
Carex illegitima Ces.	Sicily, W. Balcans Peninsula, Gavdos, W. Turkey, E. Egeo Isl., Chipre	Grecia, Attika	GCR spm0004384	W. Greuter 16872	MN761404	MN762226	MN763413	-
		Grecia, Rhodos, Laerma	GRC2 20E4ERV19	L. Schaub & J. Krause (MA893499)	MZ892157*	MZ892229*	MZ944881*	MZ892088*
Carex macrostyla Lapeyr.	Pyrenees to NW. Spain	Spain, Burgos	SPA-SP 13E6CBB18	J.A. Alejandre & M.J. Escalante (UPOS2667)	MZ892160*	MZ892231*	MZ944882*	MZ892091*
		Spain, Huesca	SPA-SP2 5E3CBB19	M. Luceño (UPOS5750)	MZ892159*	MZ892230*	MZ892024*	MZ892090*
Carex oedipostyla Duval-Jouve	Canary Isl., SW. Europe, NW. Morocco	Canary Isl., Tenerife	CNY 21E6CBB18	K. Lewejohann (GOETO19933)	MZ892166*	-	MZ892029*	MZ892096*
		Morocco, Tanger	MOR-MO 7E3CBB19	J. Fernández-Arroyo et al. (UPOS2121)	MZ892167*	MZ892236*	MZ892030*	MZ892097*
		Spain, Barcelona	SPA-SP 22E6CBB18	P. Jiménez-Mejías, S. Martín-Bravo, E. Maguilla & M. Luceño (UPOS5269)	MZ892170*	MZ892239*	MZ892033*	MZ892100*
		Spain, Cádiz, Alcalá de los Gazules	SPA-SP2 8E3CBB19	E. Maguilla, T. Villaverde & M. Luceño (UPOS5127)	MZ892169*	MZ892238*	MZ892032*	MZ892099*
		Portugal, Estremadura, Serra Sintra	PT 6E3CBB19	P. Jiménez-Mejías, M. Escudero, J. Bautista-	MZ892168*	MZ892237*	MZ892031*	MZ892098*

				Serrano & M. Luceño (UPOS1571)				
Carex peregrina Link	Azores, N. Madeira, Ethiopia (Bale Mount.) to Tanzania (Meru Mount.)	Portugal, Azores, San Miguel Isl., Furnas	AZO 4E3CBB19	S. Martín-Bravo & L. Bellón (UPOS6513)	MZ892173*	-	MZ892036*	MZ892103*
		Portugal, Madeira	MDR 12E6CBB18	M. Luceño & P. Vargas (UPOS142)	MZ892174*	MZ892242*	MZ892037*	MZ892104*
Carex phyllostachys C.A.Mey.	SE Italy, SE Albania to Greece, Turkey to N. Irán	Turkey, Prov. Adana	TUR spm0005164	Davis & Hedge 26885 (BM 000059251)	AH012956	AH012956	-	-
Carex pulicaris L.	Europe	Scotland, Highlands	GRB 19E6CBB18	S. Martín-Bravo, P. Jiménez-Mejías & M. Luceño (UPOS6291)	MZ892182*	MZ892250*	MZ892045*	MZ892112*
		Spain, Huesca, Panticosa	SPA-SP 3E3CBB19	L.E. Vendrell (UPOS3226)	MZ892183*	-	MZ892046*	-
Sect. ***Schoenoxiphium*** (Nees) Baillon								
Carex badilloi Luceño & Márquez-Corro	KwaZulu-Natal	South Africa	10E6EMS13	E. Maguilla 76EMS12 et al. (UPOS)	KY322248	KY322201	KY322360	KY322285
Carex basutorum (Turrill) Luceño & Martín-Bravo	Lesotho	South Africa	2E7EMS13	Gertenbach & Groenewald 9047 (PRE-812904)	KY322260	-	KY322376	KY322317
Carex bolusii Luceño & Márquez-Corro	Free State to Lesotho	South Africa	12E6EMS13	E. Maguilla 77EMS12 et al. (UPOS) / M. Luceño 94ML08 et al. (UPOS)	KY322247	KY322206	KY322359	KY322283
Carex burkei (C.B.Clarke) Luceño & Martín-Bravo	Cape Town to Lesotho	South Africa	12E2SMB11	J. P. H. Acock 16569 (BM) / M. Luceño 41ML10 et al. (UPOS)	KY322269	KY322172	KY322365	KY322296
Carex capensis Thunb.	Cape Town	South Africa	14E5TVH13	E. Estehmysen 33907 (BOLUS-45532)	KY322253	KY322166	KY322367	KY322300
Carex distincta (Kukkonen) Luceño & Martín-Bravo	Lesotho to KwaZulu-Natal	South Africa	14E1SMB11	B. Gehrke BG574 & M. Pirie (UPOS4390)	-	KY322188	-	-

Carex esenbeckiana Boeckeler	Ethiopia to South Africa	South Africa	4E5TVH13	E. Maguilla 51EMS12 et al. (UPOS)	KY322263	KY322178	KY322379	KY322325
Carex killickii Nelmes	South Africa	South Africa	5E5TVH13	E. Maguilla 81EMS12 et al. (UPOS)	KY322266	KY322183	KY322383	KY322332
Carex kukkoneniana Luceño & Martín-Bravo	Lesotho to KwaZulu-Natal	Malaui	4E5SMB08	B. Gehrke BG-Af321 & H. I. Patel (Z-39364)	-	KY322200	KY322362	-
Carex lancea (Thunb.) Baill.	Cape Town	South Africa	8E7EMS13	T. M. Salter 9631 (BM)	KY322273	KY322159	KY322392	KY322348
Carex ludwigii (Hochst.) Luceño & Martín-Bravo	SW. Tanzania to South Africa	South Africa	6E5TVH13	E. Maguilla 64EMS12 et al. (UPOS)	KY322246	KY322192	KY322357	KY322280
Carex multispiculata Luceño & Martín-Bravo	South Africa and Madagascar	South Africa	9E5TVH13	E. Maguilla 66EMS12 et al. (UPOS)	KY322274	KY322163	KY322394	KY322350
Carex perdensa (Kukkonen) Luceño & Martín-Bravo	South Africa	South Africa	2E5TVH13	E. Maguilla 73EMS12 et al. (UPOS)	KY322259	KY322215	KY322375	KY322316
Carex pseudorufa Luceño & Martín-Bravo	KwaZulu-Natal	South Africa	1E5TVH13	E. Maguilla 85EMS12 et al. (UPOS)	KY322245	KY322190	KY322356	KY322279
Carex schimperiana Boeckeler	Arabian Peninsula, Etiophia to South Africa	South Africa	17E5TVH13	E. Maguilla 69EMS12 et al. (UPOS)	KY322256	KY322242	KY322370	KY322304
Carex schweickerdti (Merxm. & Podlech) Luceño & Martín-Bravo	South Africa	South Africa	20E4SMB11	S. Martín-Bravo 189SMB08 et al. (UPOS)	-	KY322155	-	-
Carex spartea Wahlenb.	Madagascar, Uganda a Sudáfrica	South Africa	8E6EMS13	E. Maguilla 70EMS12c et al. (UPOS)	KY322272	KY322224	KY322391	KY322347
**Outgroup**								
Carex arctogena Harry Sm.	S. South America, Subantarctic to W. EEUU	Canada, Quebec, Argentina, Tierra del Fuego		M. J. Waterway 2003.033 (MTMG) / S. Martín-Bravo 40SMB10 et al. (UPOS4271)	KU377551	KP984465	KP996361	KP996445

Carex baldensis L.	C. & E. Alps	Switzerland, Montreaux /Italy, Bergamo, Passo della Presolana		Reznicek 8250 (MICH)/ M. Luceño & F. J. Fernández, 5ML07 (UPOS3325)	EF363121	GU176152	MN762986	KP273780
Carex canescens L.	Worldwide	Australia, Nueva Gales del Sur / Rumanía, Montes Cárpatos		M. J. Waterway 2004.147 (MTMG) / M. Puscas	KP980213	KP980398	KP980028	KR827139
Carex curvula ssp. *rosae* Gilomen	Pyrenees and Alps	France, Col du Galibier/ Spain, Huesca, Benasque		Playford 9803 et al., (FHO)/ P. Jiménez-Mejías et al. (UPOS2106)	AH012963	AH012963	-	GU176256
Carex dissitiflora Franch.	Kurils Isl., Japan, Taiwan	Japan, Honshu, Prefecture of Kyoto		Bartholomew 250 & Boufford	KP273606	KP273640	KP273680	KP273791
Carex flava L.	Europa al NW. de Irán, Canadá, NC. & NE. de EEUU	Belgium/Luxemburg/Norway		M. Luceño & M. Guzmán (UPOS403) / M. Leten	KU939525	JN634682	KU939681	JN627772
Carex gibba Wahlenb.	Japan, Korea to Vietnam	China, Shaanxi, Yang Xian		Tsugaru 17908 (MO) / Zhu 2776 et al. (MO)	EU001138	EU000996	KP273685	KP273796
Carex hypolytroides Ridl.	Indochina, C. Sumatra, N. Borneo	Vietnam, Kon Tum, NW de Ngoc Linh		J. R. Starr STAR679	KP273612	KP273647	KP273690	KP273800
Carex meridensis (Steyerm.) J.R. Starr	NW. Venezuela to Subantarctic Isl.	Ecuador, Pichincha province		Starr 99028 & Amigo (FHO) / M. Luceño & R. Álvarez (UPOS1810)	AY244536	GU176170	-	GU176269
Carex siderosticta Hance	Russia, China, Japan	Japan, Honshu, Gunma Prefecture / Canada, Quebec, Gatineau		M. J. Waterway 2004.268 (MTMG) / Starr (CAN)	DQ998892	DQ998946	KU496588	KP273817
